# Pathological BBB Crossing Melanin-Like Nanoparticles as Metal-Ion Chelators and Neuroinflammation Regulators against Alzheimer’s Disease

**DOI:** 10.34133/research.0180

**Published:** 2023-06-23

**Authors:** Qianqian Huang, Chaoqing Jiang, Xue Xia, Yufan Wang, Chenxing Yan, Xiaorong Wang, Ting Lei, Xiaotong Yang, Wenqin Yang, Guo Cheng, Huile Gao

**Affiliations:** ^1^ Key Laboratory of Drug-Targeting and Drug Delivery System of the Education Ministry, West China School of Pharmacy, Sichuan University, Chengdu 610041, P.R. China.; ^2^ Laboratory of Molecular Translational Medicine, Center for Translational Medicine, Key Laboratory of Birth Defects and Related Diseases of Women and Children (Sichuan University), Ministry of Education, Department of Pediatrics, West China Second University Hospital, Sichuan University, Chengdu 610041, P.R. China.

## Abstract

Inflammatory responses, manifested in excessive oxidative stress and microglia overactivation, together with metal ion-triggered amyloid-beta (Aβ) deposition, are critical hallmarks of Alzheimer’s disease (AD). The intricate pathogenesis causes severe impairment of neurons, which, in turn, exacerbates Aβ aggregation and facilitates AD progression. Herein, multifunctional melanin-like metal ion chelators and neuroinflammation regulators (named PDA@K) were constructed for targeted treatment of AD. In this platform, intrinsically bioactive material polydopamine nanoparticles (PDA) with potent metal ion chelating and ROS scavenging effects were decorated with the KLVFF peptide, endowing the system with the capacity of enhanced pathological blood–brain barrier (BBB) crossing and lesion site accumulation via Aβ hitchhiking. In vitro and in vivo experiment revealed that PDA@K had high affinity toward Aβ and were able to hitch a ride on Aβ to achieve increased pathological BBB crossing. The engineered PDA@K effectively mitigated Aβ aggregate and alleviated neuroinflammation. The modulated inflammatory microenvironment by PDA@K promoted microglial polarization toward the M2-like phenotype, which restored their critical functions for neuron care and plaque removal. After 3-week treatment of PDA@K, spatial learning and memory deficit as well as neurologic changes of FAD^4T^ transgenic mice were largely rescued. Transcriptomics analysis further revealed the therapeutic mechanism of PDA@K. Our study provided an appealing paradigm for directly utilizing intrinsic properties of nanomaterials as therapeutics for AD instead of just using them as nanocarriers, which largely widen the application of nanomaterials in AD therapy.

## Introduction

Alzheimer’s disease (AD), the most common type of dementia, is a chronic, age-related, multifactorial neurodegenerative disease causing progressive cognitive and memory deterioration [1,2]. In 2019, Alzheimer’s Disease International estimated that AD had a devastating impact on more than 50 million people globally and is expected to triple in 2050 [3,4], which not only brings severe suffering for patients and caregivers, but also gives rise to a huge economic burden on society [5,6]. Currently, clinical therapeutic agents for AD treatment using acetylcholinesterase inhibitors (donepezil, rivastigmine, and galantamine) and non-competitive *N*-methyl-d-aspartate receptor modulator (memantine) only palliatively relieve cognitive and behavior deterioration but do not prevent further disease progression [7,8]. Hence, efficient therapies targeting pathological mechanisms of AD are imperative.

AD pathogenesis is believed to be mutually reinforced by multiple complex mechanisms [9–12]. Recent advances indicated that neuroinflammation is evident in the early stage of AD patients owing to exacerbated immune response [13,14]. As the important initiator of neuronal damage, oxidative stress leads to more Aβ accumulation through upregulation of the amyloid precursor protein (APP) and increased secretase activity [13,15,16]. In turn, aberrant aggregation of Aβ further exhibits neurotoxicity and triggers pro-inflammatory response [17]. Remodeling abnormal microenvironment of AD lesion by reactive oxygen species (ROS) cascade elimination will be a new perspective for early AD therapy [18–20]. In AD brain, the imbalance of metal ions also contributes to AD development [12,21,22]. Metal ions such as Cu^2+^ and Zn^2+^ greatly accelerate neurotoxic Aβ plaque deposition due to high affinity with the N terminus of Aβ [23–25]. The abnormally high concentration of metal ions within Aβ deposits not only stabilizes neurotoxic Aβ plaques but also catalyzes ROS formation and exacerbates neuroinflammation [26,27]. Metal-ion chelation has proven to be an effective strategy for AD therapy to prevent Aβ deposition and ROS production [28–30]. Since limited effectiveness toward single Aβ inhibition to regulate cognitive decline have been shown in numerous trials [31,32], multi-target strategies in AD therapy would be more promising.

As intrinsic immune cells of the brain, microglia are precisely tailored by brain environment [33]. Mounting evidence showed that microglial dysfunction initiates at a very early stage and persists throughout the whole AD development [14,34]. What is worse, the abnormal function of microglia intertwines with Aβ deposits and jointly promote AD aggravation. Specifically, the accumulated Aβ plaques impair phagocytic function of microglia and transform microglia to a pro-inflammatory M1 phenotype, resulting in overproduction of ROS and various inflammatory factors [35,36], which, in turn, undoubtedly promote the production and aggregation of Aβ [37,38]. The vicious crosstalk between dysfunctional microglia and Aβ further aggravates AD progression. Taken together, reversing neuroinflammation microenvironment and modulating activated microglia as well as reducing Aβ burden synchronously by ROS elimination and metal ion chelation is a promising multitasking strategy to break the feedback between Aβ and neuroinflammation for AD therapy.

Functional nanomaterials are emerging as new favorites as their smart utilization simplifies the entire drug delivery system while broadening functionality [39–41]. Very convincing evidence now supported the idea that functional nanomaterials show great potential as promising tools for AD treatment [35,42,43]. Although therapeutic agents can be delivered into the brain with the help of a nano size effect that increases blood–brain barrier (BBB) penetration [44,45], the AD locus selectivity was unsatisfactory. It was reported that periphery Aβ could be easily internalized into the brain mediated by the highly expressed advanced glycation end-product (RAGE) receptor in AD brain, which is closely related to the pathogenesis of neuronal dysfunction and death in AD [46,47]. A molecular modeling study indicated that RAGE interacts with the N-terminal of Aβ and transports Aβ from the blood to the brain [48]. Inspired by the transportation of Aβ, KLVFF peptide, derived from Aβ without interfering with normal interactions, was reported to be capable of acting as “a binding element” for selectively targeting the peripheral Aβ in AD patients [49,50]. Hence, utilization of Aβ peptide modification in AD therapy may be a promising strategy for enhancing pathological BBB penetration and lesion accumulation by Aβ hitchhiking.

**Fig. 1.**
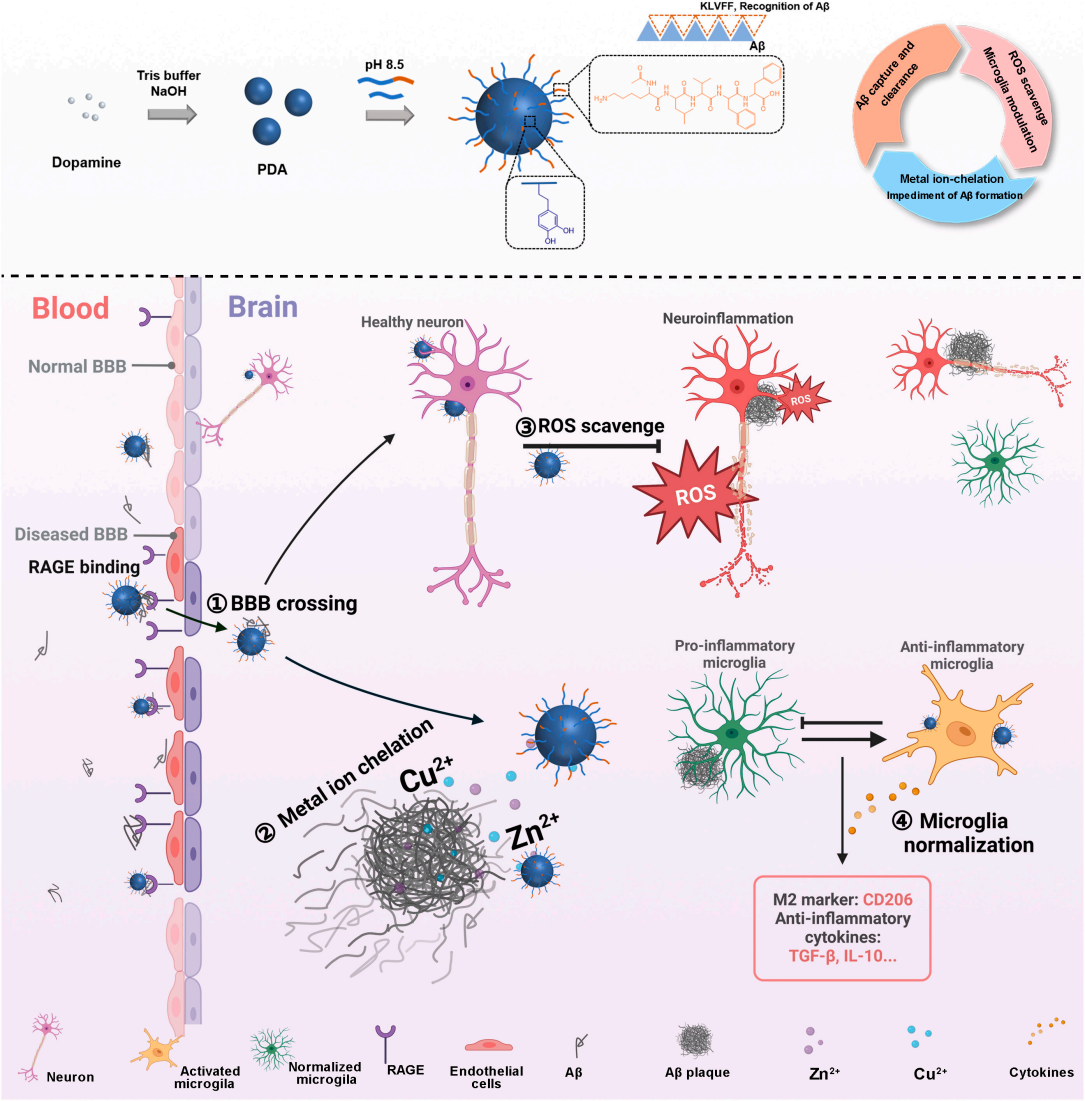
Schematic diagram of the strategy.

Recently, dopamine-derived melanin-based polydopamine nanoparticles (PDA), which are highly biologically relevant to natural melanin, have been suggested as safe and potent antioxidants and metal chelators in biomedical applications [51,52]. To our knowledge, melanin-like nano-antioxidants or metal chelators have not been investigated in AD applications. Herein, based on complex etiologies in AD development, novel melanin-like metal ion chelators and neuroinflammation regulators (PDA@K) that reduce Aβ burden and normalize microglia dysfunctional synergistically through metal chelation and ROS scavenging were designed for AD therapy (Fig. 1). Given the low enrichment at the lesion site, PDA were modified with KLVFF peptides. PDA containing KLVFF could selectively target the peripheral Aβ in the AD model and achieved enhanced pathological BBB penetration by Aβ hitchhiking via highly expressed RAGE receptors. Once they enter the brain, PDA@K bind to amyloid plaques and greatly enriched at the lesion site, lowering the metal ion level and promoting amyloid plaque depolymerization. PDA@K endocytosed by lesion cells reduced ROS level and alleviated neuroinflammation. Effective Aβ targeting, pathological BBB crossing, and ROS scavenging capacity with excellent biocompatibility were demonstrated in vitro and in vivo. The modulated inflammatory microenvironment promoted microglial polarization and restored their critical functions of plaque clearance. This multifunctional strategy exhibited potent microglia modulation and neuroprotection in FAD^4T^ transgenic mice, with improvement in memory and cognitive impairment. Our work proved the validity by directly using nanomaterials as anti-AD drugs, which, together with the prospect as biosafe nanocarriers for clinical AD drug delivery, greatly widen the application of nanomaterials for AD therapy.

## Results

### Preparation and characterization of NPs

**Fig. 2.**
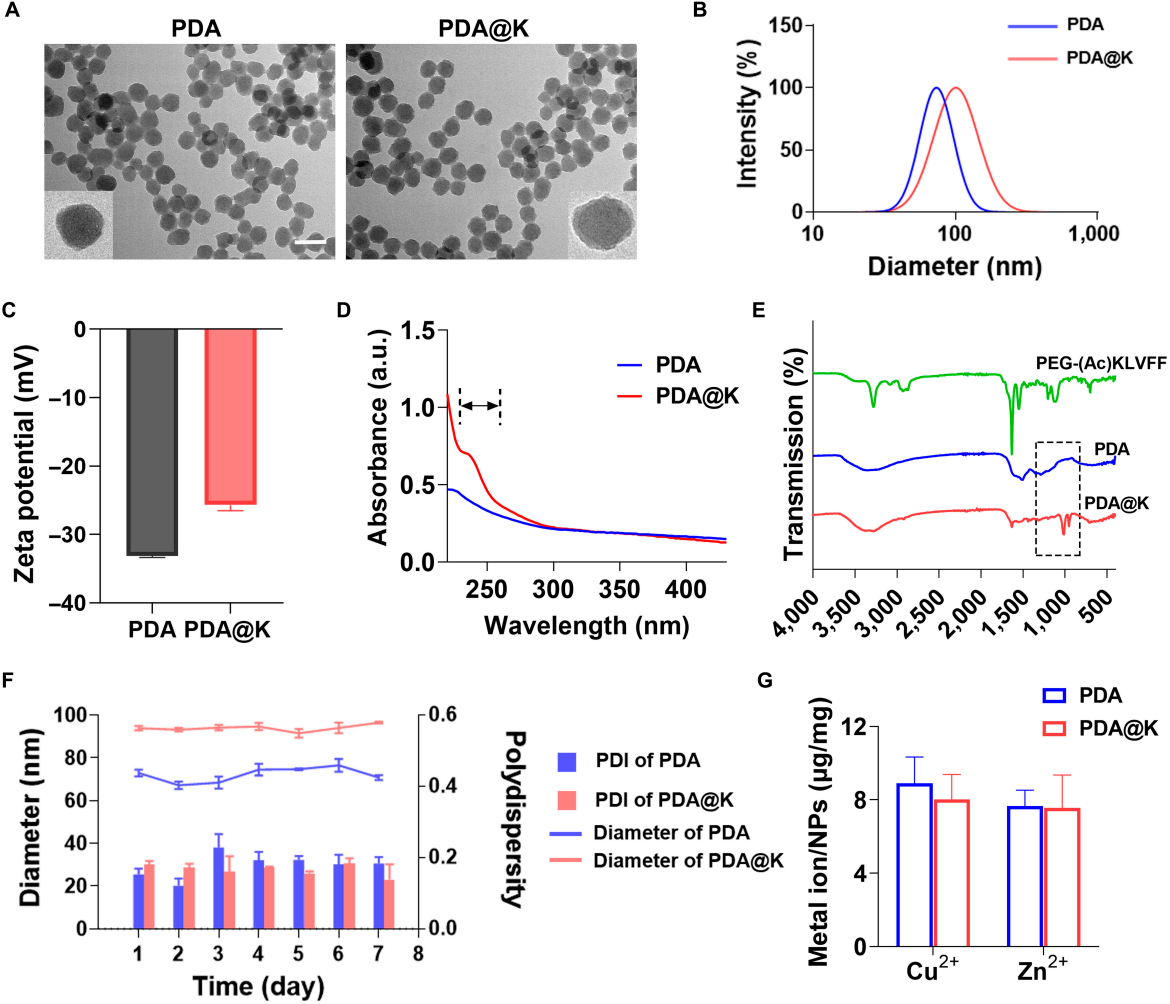
Synthesis and characterization of NPs. (A) The TEM images of PDA and PDA@K. Scale bar, 100 nm. (B) The particle size of PDA and PDA@K by DLS analysis. (C) Zeta potentials of PDA and PDA@K. (D) UV spectra of NPs with KLVFF and without KLVFF sequences. (E) FTIR spectra of PEG-_(Ac)_KLVFF, PDA, and PDA@K, respectively. The peak circled in black box is attributed to the C-O-C stretching of PEG on the surface of PDA@K. (F) Changes in particle sizes of NPs and PDI in ultrapure water during incubation for 7 days at 37 °C. (G) Cu^2+^ and Zn^2+^ binding capability of PDA and PDA@K measured by ICP-OES and AAS. All data are presented as mean ± SD, *n* = 3.

PDA were fabricated using a classic method and optimum particle size was obtained by adjusting the pH of the reaction. Figure 1 elucidates the rational synthesis route as well as their further modification with Aβ-recognition peptide, abbreviated as PDA@K. Images from transmission electron microscopy (TEM) indicated that the PDA and PDA@K held monodispersed spherical structures with an average diameter of about 60 nm (Fig. 2A). After modification, the hydrodynamic size of PDA@K measured by dynamic light scattering (DLS) was about 90 nm (Fig. 2B). The minuscule particle size and narrow size distribution were beneficial for BBB penetration [53]. The zeta potentials of synthesized PDA and PDA@K were −33.10 ± 0.26 mV and −25.70 ± 0.82 mV, respectively (Fig. 2C). Considering strong ultraviolet (UV)–visible adsorption at 250 to 260 nm of phenylalanine residues (Phe, F, Fig. S3) [54], the UV spectrum of PDA@K and PDA was measured to verify the modification of the NH_2_-PEG-_(Ac)_KLVFF chain. The much higher UV absorption of PDA@K at 250 to 300 nm than that of PDA confirmed the successful integration of KLVFF fragments (Fig. 2D). The C-O-C stretching of PEG in the Fourier transform infrared spectroscopy (FTIR) spectrum further verified the successful modification of the KLVFF peptide chain (Fig. 2E). Hydrodynamic diameters of these NPs were measured by DLS and showed no obvious changes for 7 days (Fig. 2F), indicating their good stability. After that, the metal ion chelating ability of PDA and PDA@K was tested using an inductively coupled plasma-optical emission spectrometer (ICP-OES) and an atomic absorption spectrophotometer (AAS). KLVFF modification did not affect the chelating ability of NPs to metal ions. PDA and PDA@K all showed slightly different binding properties for Cu^2+^ (8.91 ± 1.17 μg/mg vs. 8.03 ± 1.11 μg/mg) and Zn^2+^ (7.66 ± 0.71 μg/mg vs. 7.56 ± 1.46 μg/mg) under the same conditions (Fig. 2G). In AD brains, the accumulation of copper and zinc observed was 5.7 and 3.1 times higher than that in normal brains, which clearly influenced Aβ aggregation [55]. The excellent Cu^2+^ and Zn^2+^ chelating ability of PDA offers great potential for AD therapy.

### In vitro ROS scavenging capacity

**Fig. 3.**
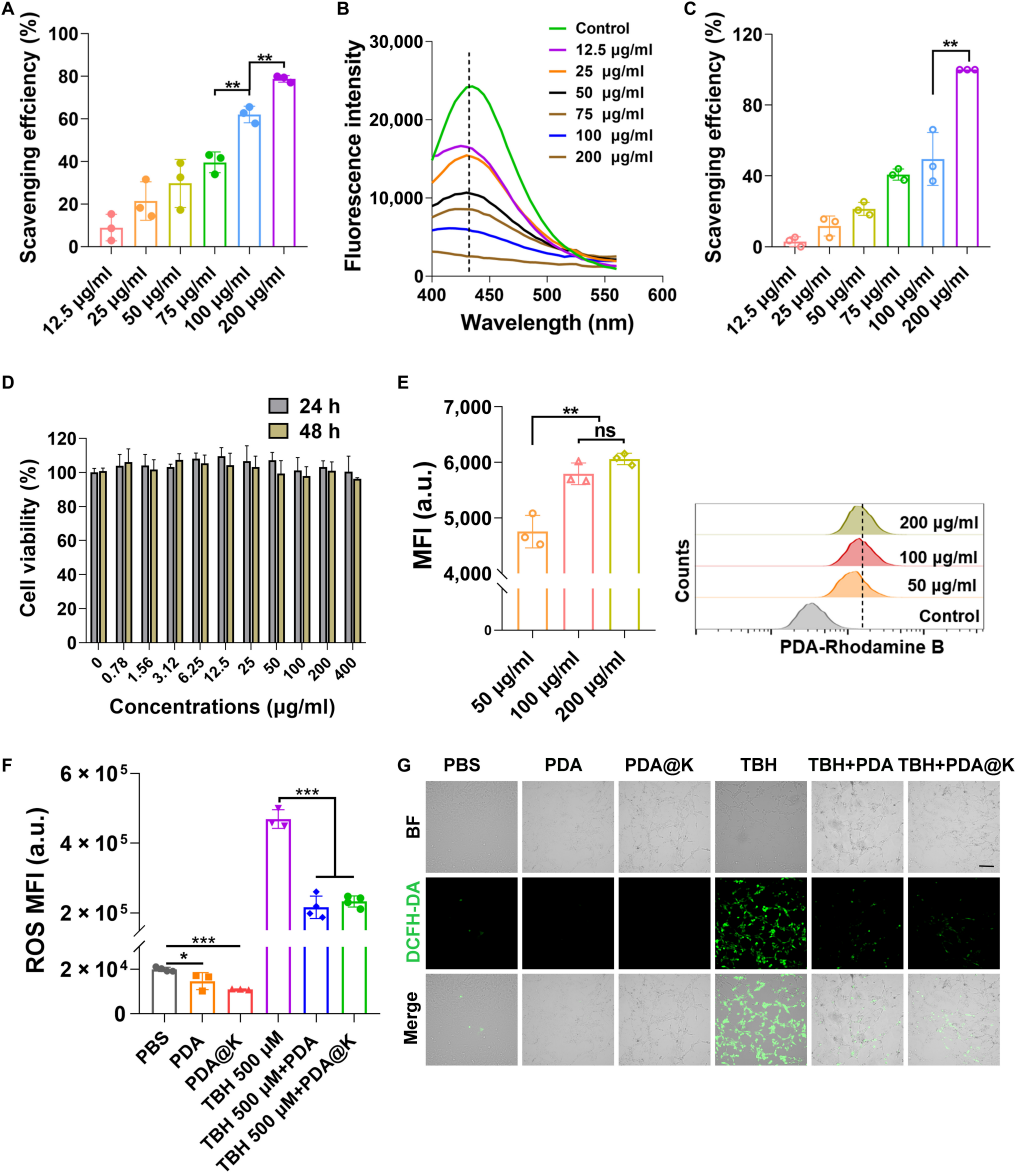
In vitro ROS scavenging capacity. ROS scavenging efficiency of PDA for O_2_•^−^ (A), •OH (B), and H_2_O_2_ (C). (D) Cell viability of PC-12 cells after incubation with different concentrations of PDA for 24 h and 48 h measured by MTT assay, respectively. (E) Cellular uptake of PDA by PC-12 via flow cytometry (FCM) analysis. Representative FCM histograms are shown on the right. Intracellular ROS is detected by (F) flow cytometry and observed via (G) a fluorescence microscope. Scale bar, 100 μm. Data are presented as mean ± SD, *n* = 3. *P < 0.05, **P < 0.01, ***P < 0.001. MFI, mean fluorescence intensity.

Considering the crucial role of ROS in the prevalence of AD, the scavenging capability of PDA to consume ROS was investigated systematically because of rich catechol structures on the PDA surface. Hydroxyl radicals (•OH), superoxide radicals (O_2_•−), and hydrogen peroxide (H_2_O_2_) were chosen to react with PDA to test their multiple ROS scavenging capability. As shown in Fig. 3A, PDA were highly sensitive to O_2_•−, and successfully scavenged 62.04 ± 3.86 % of O_2_•− at a concentration of 100 μg/ml. As PDA concentration increased, a concentration-dependent scavenging behavior toward O_2_•− was observed. •OH removal efficiency was evaluated by measuring the fluorescent signal of 2-hydroxyterephthalic acid. A dramatic decrease in fluorescence intensity (FI) around 425 nm was detected in the presence of PDA with different concentrations, confirming that PDA could efficiently eliminate •OH (Fig. 3B and Fig. S6). Since H_2_O_2_ had strong UV absorption at 240 nm, H_2_O_2_ scavenging ability was directly monitored by UV spectrum. The high fit (*r* = 0.9998) of H_2_O_2_ concentrations with UV signals at 240 nm verified the sensitivity of this approach (Fig. S7). After incubation with PDA, the UV peaks around 240 nm diminished gradually with increasing NP concentrations, which showed enhanced scavenging efficiency (Fig. 3C). In brief, the scavenging efficiency of PDA toward O_2_•−, •OH, and H_2_O_2_ all followed a concentration-dependent manner. These results confirmed admirable multiantioxidative activities of PDA toward various ROS in vitro.

Prior to the investigation of intracellular ROS removal ability of NPs, cytotoxicity and uptake kinetics toward NPs were essential parameters. Standard methyl thiazolyl tetrazolium (MTT) assay was carried out to determine viabilities of PC-12 cells toward PDA and PDA@K. No significant cytotoxicity induced by PDA and PDA@K was observed for all the groups (24 h and 48 h) even at a high concentration of 400 μg/ml (Fig. 3D and Fig. S8). Cellular uptake of PDA greatly increased at 100 μg/ml. Since there was no significant difference in cellular uptake between 100 μg/ml and 200 μg/ml, subsequent in vitro experiments were performed at 100 μg/ml (Fig. 3E). We further examined the protective effect of PDA and PDA@K on cells against ROS damage. PC-12 cells were pretreated with tert-butyl hydroperoxide (TBH) to mimic oxidative stress conditions. The FI of ROS^+^ cells, increased by TBH treatment (from 0.89% to 75.19%), was significantly reduced by the treatment of PDA and PDA@K (to 37.10% and 36.84%, respectively). PDA and PDA@K not only reduced ROS levels of positive control cells, but also significantly reduced cellular basal ROS levels (Fig. 3F and Fig. S9). The same conclusion was obtained from fluorescence microscopy (Fig. 3G). The ROS scavenging capacity of PDA was not affected after KLVFF modification. After 2 h of TBH treatment, cells became round and floated up, showing a dead state, while PDA and PDA@K treatment effectively prevented these damages. The cell morphology in TBH+PDA and TBH+PDA@K groups was the same as that of the phosphate buffered saline (PBS) group (Fig. S10). Taken together, PDA and PDA@K emerged as high-performance ROS scavengers for neuroprotection.

### Inhibition of Aβ aggregation and disaggregation effects toward Aβ fibrils

**Fig. 4.**
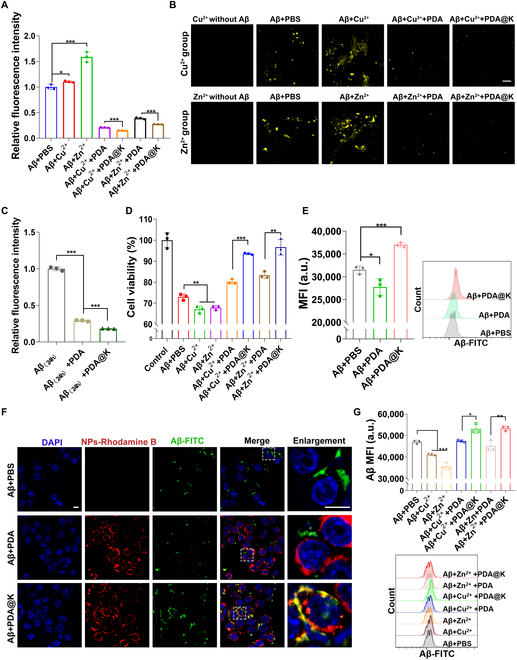
Inhibition and disaggregation effects toward Aβ, anti-Aβ toxicity, and Aβ clearance. (A) The relative fluorescence intensity of Aβ_42_ monomers incubated with or without different NPs and metal ions for 24 h measured by a microplate reader. (B) ThT fluorescence images in (A). Scale bar, 50 μm. (C) The relative fluorescence intensity of Aβ fibrils incubated with NPs for disaggregation experiments. Aβ_42_ was preincubated with PBS at 37 °C for 24 h before incubation with NPs (Aβ_(24h)_). (D) The anti-Aβ toxicity ability of PDA and PDA@K analyzed in PC-12 cells. (E) Cell uptake of Aβ by BV2 cells after treatment with different NPs. Aβ_42_ was labeled with FITC. Representative FCM histograms are shown on the right. (F) Co-localization analysis of Aβ_42_ and NPs in BV2 cells (blue: DAPI; green: Aβ; red: NPs). Scale bars represent 10 μm. (G) Cell uptake of Aβ_42_ by BV2 cells when co-incubated with different NPs and metal ions. Representative FCM histograms are shown below. Data are presented as mean ± SD, *n* = 3. *P < 0.05, **P < 0.01, ***P < 0.001. MFI, mean fluorescence intensity.

The excellent Cu^2+^ and Zn^2+^ chelating ability of PDA has been described in the previous section. Given the crucial roles of Cu^2+^ and Zn^2+^ in Aβ aggregation [23,56], the aggregation kinetics of Aβ_42_ were observed via thioflavin T (ThT) assay. The rapid increase of ThT FI with increasing metal ion concentrations confirmed the promoting effect of metal ions on Aβ aggregation (Fig. S11). ThT signals recorded in inhibition experiments indicated that NPs inhibited amyloid aggregate formation. Almost 90% reduction in ThT FI was measured when Aβ_42_ was cocultured with metal ions and PDA@K compared to the group added with metal ions alone (Fig. 4A). With the addition of NPs, a large number of preformed Aβ_42_ complexes were transformed into puncta (Fig. 4B). In disaggregation experiments, NPs were added into Aβ_42_ fibrils to verify their de-crosslinking effects. Under incubation with NPs, the ThT FI was remarkably decreased (Fig. 4C). The average FI in PDA groups was 29%, and the degree of fluorescence quenching was more pronounced in all the PDA@K groups, with only 19% remaining. All these results indicated that PDA@K exhibited splendid Aβ fibril inhibition and disaggregation effects in vitro. Considering the presence of serum proteins and a small amount of metal ions in vivo, we tested whether serum would affect the metal chelating ability of NPs. The in vitro results showed that incubation with serum for 24 h did not affect their inhibitory effect on Aβ_42_ aggregation in the presence of metal ions, laying the foundation for their application in vivo (Fig. S12). Aβ oligomers induce serious neurotoxicity by damaging hippocampal neurons through multiple mechanisms [57,58]. We then verified whether inhibiting Aβ_42_ aggregation could reduce the toxicity caused by Aβ at the cellular level. PC-12 cells were treated with Aβ_42_ (20 μmol/l), metal ions, and different NPs. Compared with those treated with Aβ_42_/PBS, the addition of metal ions further reduced cell viability, but increased when co-incubated with NPs, especially in the PDA@K group. Cell survival rate all surpassed 90% after incubating with PDA@K (Fig. 4D). These results together indicated that NPs, especially PDA@K, could effectively reduce Aβ-induced toxicity by inhibiting Aβ aggregation through metal chelation.

### Evaluation of in vitro Aβ capturing

As resident macrophages of the brain, microglia have been shown to play a central role in Aβ clearance [59–61]. Herein, murine microglia BV2 was employed to evaluate the cellular uptake of Aβ. To check the targeting effect of PDA@K toward Aβ, BV2 cells were incubated with fluorescein-5-isothiocyanate (FITC)-labeled Aβ_42_ and Rhodamine B (RB)-labeled NPs. The uptake of Aβ_42_ by BV2 was studied via flow cytometry (FCM). As shown in Fig. 4E, a strong increase in FITC-labeled Aβ_42_ signal within BV2 cells was observed after co-culture with PDA@K, suggesting that KLVFF-modified PDA were capable of capturing Aβ_42_ and promoting microglia uptake toward Aβ_42_. From the co-localization study, it was found that the green signals emitted by Aβ_42_ and the red signals emitted by PDA@K were almost totally co-localized in BV2 cells. Free Aβ cannot be easily endocytosed by microglia. This verified that PDA@K recognized Aβ_42_ and formed NPs-Aβ_42_ complexes, facilitating microglia to restore the capability of Aβ_42_ phagocytosis (Fig. 4F). Inspired by these promising results, we then evaluated the ability of NPs to promote Aβ phagocytosis by microglia in the presence of metal ions. As expected, after incubation of Aβ_42_ with metal ions, Aβ_42_ phagocytosis by BV2 significantly decreased since Aβ_42_ aggregation formed, but with the help of NPs, especially PDA@K, the uptake of Aβ_42_ significantly increased (Fig. 4G). The ThT and fluorescent microscope together with FCM results revealed that PDA@K could not only inhibit Aβ aggregation by their metal-chelating ability but also capture disaggregated Aβ and form NPs/Aβ complex to promote microglial uptake, which greatly reduce extracellular Aβ deposition and Aβ-induced toxicity.

### NPs suppressed inflammation and reprogrammed the microenvironment to enhance neuroprotection in vitro

**Fig. 5.**
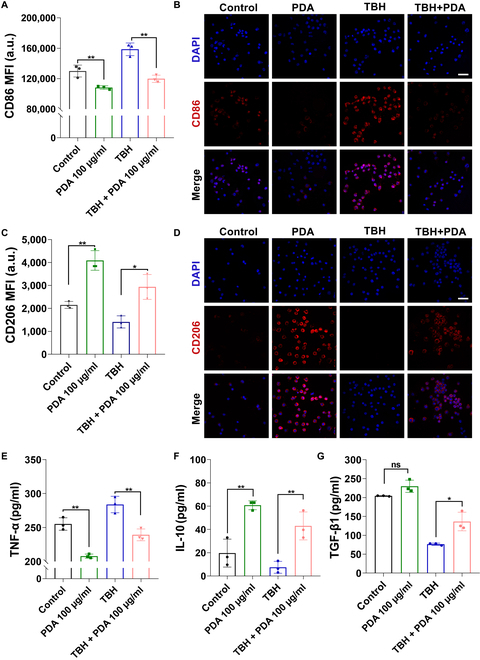
NP treatment suppressed pro-inflammatory responses of microglia by inducing polarization toward the M2 phenotype. (A) CD86 expression measured via flow cytometry. TBH treatment was used as positive controls. (B) Representative confocal images for CD86 expression analysis. Scale bar, 50 μm. (C) CD206 expression measured using flow cytometry. (D) Representative confocal images for CD206 expression analysis. Scale bar, 50 μm. Enzyme-linked immunosorbent assay (ELISA) results showing that NPs prevented the TBH-induced increase of pro-inflammatory factor TNF-α (E) and reversed the decrease of anti-inflammatory factors IL-10 (F) and TGF-β (G). Data are presented as mean ± SD, *n* = 3. *P < 0.05, **P < 0.01, ***P < 0.001. MFI, mean fluorescence intensity.

Microglia can be protective (M2) or detrimental (M1) under different circumstances [62]. In AD brain, the deposition of Aβ is accompanied by the production of ROS, which leads to an inflammatory environment and promotes microglia dysfunction [63,64]. Improvement of the inflammatory environment contributes to microglia normalization. To determine whether PDA treatment could reprogram microglia toward the M2 phenotype, primary BV2 cells were first activated with TBH to mimic the inflammatory environment and verified via FCM. The expression of the M1-type marker CD86 increased with increasing TBH concentrations, indicating that the inflammatory environment indeed disturbed microglia homeostasis (Fig. S14). FCM and immunofluorescence staining results showed that PDA inhibited the activation of M1 microglia as characterized by the reduced expression of CD86 (M1 pro-inflammatory marker) (Fig. 5A and B). By contrast, they promoted the polarization of M2-type microglia as indicated by the increased expression of CD206 (M2 anti-inflammatory marker) (Fig. 5C and D). In the pro-inflammatory M1 state, microglia secrete pro-inflammatory cytokines, which further cause ROS accumulation and neuronal apoptosis. When transformed to an anti-inflammatory state, M2 microglia enhance neural regeneration by producing beneficial neurotrophins and anti-inflammatory cytokines [62]. Benefitting from the satisfactory polarization capability of PDA, we further observed the downregulation of typical pro-inflammatory cytokine (TNF-α) and the upregulation of anti-inflammatory cytokines (IL-10 and TGF-β) in BV2 cells treated with PDA/PDA+TBH compared with the PBS/TBH group (Fig. 5E to G). These results demonstrated that the PDA core could promote the polarization of microglia into the neuroprotective M2-like phenotype.

### Pathological BBB crossing and intracerebral distribution of NPs

**Fig. 6.**
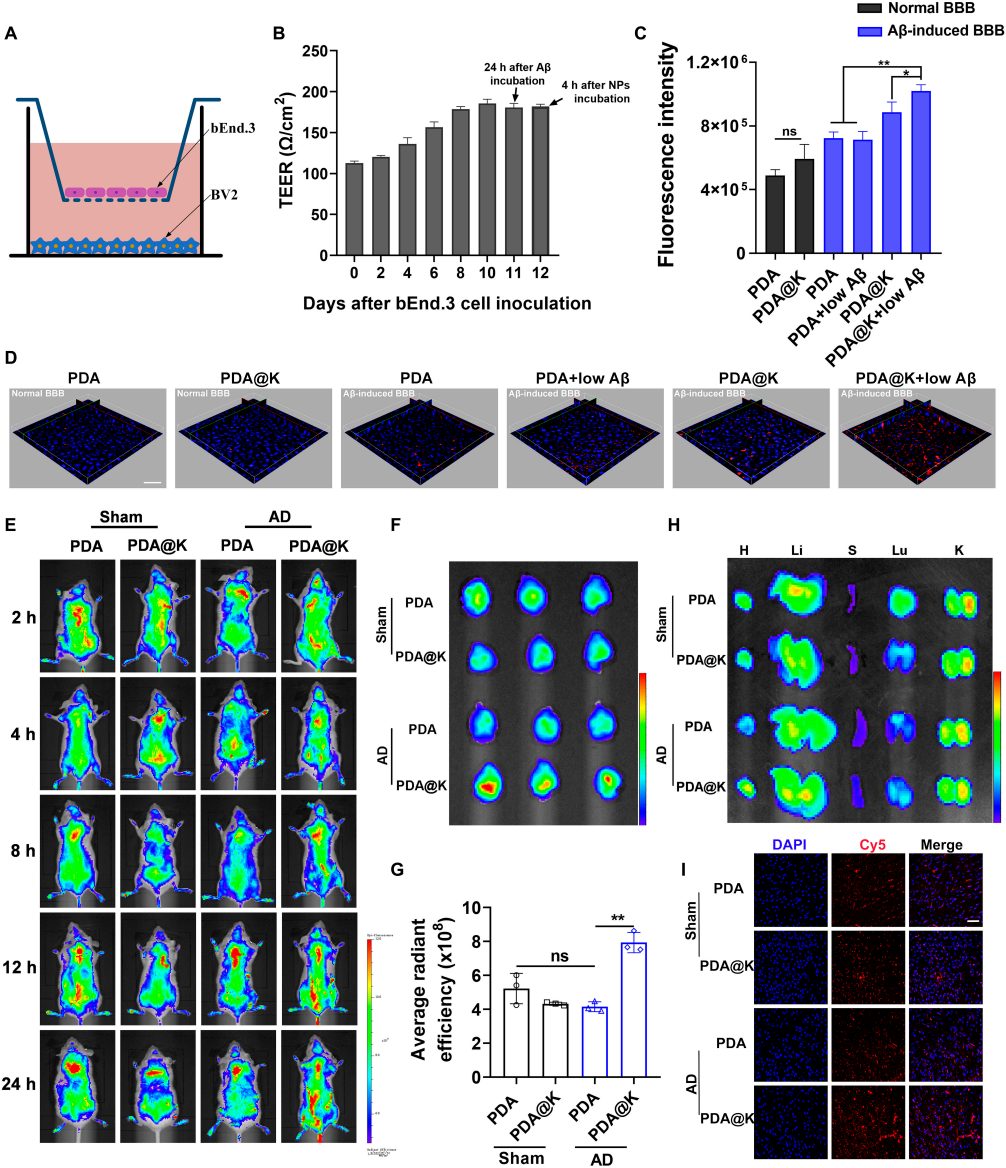
Pathological BBB crossing and intracerebral distribution of NPs. (A) The schematic illustration of the transwell BBB model. (B) The transendothelial electrical resistance (TEER) values monitoring of the bEnd.3 monolayer of the BBB model. (C) Fluorescence intensity of the supernatant liquid in the lower chamber after the introduction of NPs for 4 h. (D) 3D fluorescent images of the bEnd.3 layer (blue: DAPI; red: NPs). Scale bar, 100 μm. (E) In vivo fluorescent imaging of mice in sham operation and AD group (Aβ-injected mice) at 2, 4, 8, 12, and 24 h post tail intravenous injection with PDA and PDA@K. (F) Ex vivo fluorescent imaging and (G) Semi-quantification of brains at 24 h post-injection. (H) Ex vivo fluorescent imaging of major organs at 24 h post-injection. (I) Confocal observation of NPs distribution in brain slices at 24 h after administration. Scale bar represents 50 μm. Data are presented as mean ± SD, *n* = 3. *P < 0.05, **P < 0.01, ***P < 0.001.

Since BBB is a major challenge for brain drug delivery, BBB penetration is one of the pivotal indices of nanodrugs in clinical AD therapy [65,66]. To substantiate BBB permeability of the NPs, a normal in vitro BBB model was established by seeding bEnd.3 in the upper chamber and BV2 in the lower chamber (Fig. 6A). When the transendothelial electrical resistance (TEER) values of the bEnd.3 monolayer were greater than 180 Ω/cm^2^, the BBB barrier formed (Fig. 6B). In order to mimic the pathological condition in AD, bEnd.3 and BV2 cells in the transwell model were incubated with Aβ_42_ oligomer (5 μmol/l) for 24 h to form AD-mimetic BBB. Afterwards, low concentrations of Aβ_42_ (4.5 nM) were added to the upper chamber to simulate peripheral circulation of AD. PDA@K were first incubated with Aβ_42_ solution (4.5 nM) to check peripheral Aβ targeting of PDA@K. The decreasing fluorescence values indicated that PDA@K selectively target peripheral Aβ in a concentration-dependent manner (Fig. S15). The expression of RAGE was then examined and found to be significantly elevated in both Aβ-induced bEnd.3 cells and AD transgenic mice brains (Fig. S16). To test whether PDA@K can enhance BBB crossing by Aβ hitchhiking in AD, NPs labeled with RB were then added into the upper inserts of the normal and AD-mimetic BBB models with or without low concentration of Aβ_42_ (4.5 nM) and incubated for 4 h. In AD-mimetic BBB, significantly enhanced FI in the PDA@K+low Aβ group was detected in the lower chamber (Fig. 6C). Meanwhile, BV2 endocytosis toward PDA@K was increased in AD-mimetic BBB with the addition of low concentration of Aβ_42_ (Figs. S17 and S18). However, this phenomenon was not observed in the PDA or normal BBB model group. The 3D fluorescent images of the bEnd.3 layer showed that the PDA@K+low Aβ group was more permeable than the other groups (Fig. 6D). These results suggested that PDA containing the KLVFF peptide could specifically penetrate pathological BBB in AD lesion, at least in part, attributed to Aβ hitchhiking via highly expressed RAGE in AD.

Given the transmembrane and Aβ affinity capabilities of PDA@K, we further investigated their biodistribution and brain accumulation in healthy and AD mice, respectively. To track the in vivo circulation, NPs were labeled with Cy5 dyes (Fig. S19) and investigated via whole-body fluorescence imaging. Notably, at the same time point, fluorescence signals of PDA@K in the brain in the AD group were much stronger than the other groups, while in sham-operation mice, the brain FI of PDA and PDA@K was similar, confirming that KLVFF coating was beneficial for specific pathological BBB penetration (Fig. 6E). At 24 h post injection, mice were sacrificed and both major organs (including heart, liver, spleen, lung, and kidney) and brain tissues were isolated for ex vivo imaging. Although high level of NPs was detected in livers, the brain FI of PDA@K in AD mice was much stronger than the other groups (Fig. 6F and H). According to semi-quantification of brain fluorescence (Fig. 6G), the brain accumulation of PDA@K was 1.92 times higher than that of PDA in AD mice. Afterwards, brains and organs were frozen-sliced to further observe the accumulation of NPs by confocal imaging (Fig. 6I and Fig. S20). The brain FI of PDA@K in the AD group was stronger than that of other groups, which was basically in line with the above results. In summary, Aβ recognition by PDA@K facilitated their passage through pathological BBB and thus achieved AD brain targeting, which was in accordance with the in vitro BBB penetration results.

### Behavioral evaluation of NPs therapy in FAD^4T^ transgenic mice

**Fig. 7.**
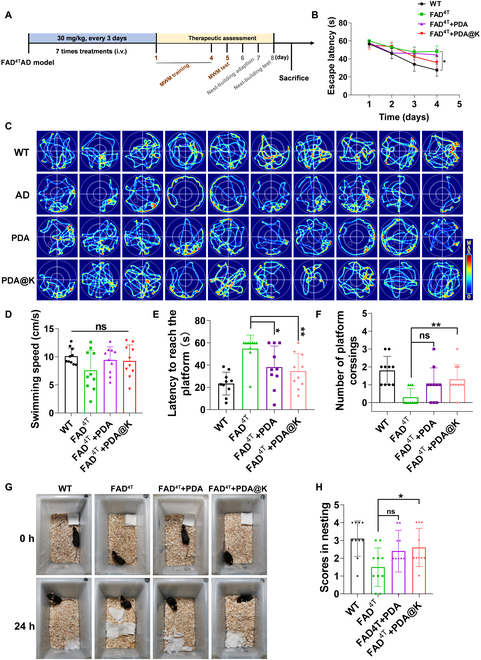
Behavioral evaluation of NPs therapy in FAD^4T^ transgenic mice. (A) Schematic diagram of the experimental timeline. FAD^4T^ transgenic and WT mice were treated with NPs or 5% glucose via tail intravenous injection every 3 days for 7 cycles. Mice were then subjected to MWM and nesting tests for memory evaluation, and samples were collected for molecular pathological assessments. (B) Escape latencies during the initial training stage of MWM. Data presented as mean ± SD, *n* = 10 mice per group, *P < 0.05. (C) The search paths of mice in the MWM test. (D) Swimming speed of mice in each group during the MWM test. (E) Time to reach the target platform and (F) the number of platform crossings in different groups after treatment. Data are presented as mean ± SD, *n* = 10 mice per group, *P < 0.05, **P < 0.01. (G) Representative images from the nest-building experiment in FAD^4T^ and WT mice. Photos were taken 24 h after the introduction of nesting material into the cage. (H) Nest-building scores for each group. The scoring criteria were described in detail in the Materials and Methods section. Data are presented as mean ± SD, *n* = 10, *P < 0.05.

To evaluate the therapeutic effect of NPs for AD, the FAD^4T^ transgenic mice model was assessed in behavioral tests to study their preventive effect on learning and memory impairment relevant to AD. The FAD^4T^ transgenic mouse is a multitransgenic AD model that expresses the Swedish and Indiana mutated APP gene and the PSEN1 gene with M146V and L286V mutations. Compared to the commonly used transgenic AD model, FAD^4T^ mice express accelerated amyloid deposition (1.5 months) and microglia activation (1.5 months) with obvious memory deficits (5 month), demonstrating suitability and reliability for evaluating the therapeutic efficacy of NPs in this novel model. According to the workflow in Fig. 7A, 5-month-old FAD^4T^ male mice were randomly grouped and injected with NPs through the tail vein 7 times every 3 days. Five percent glucose-injected FAD^4T^ and control wild-type (WT) mice groups were included to assess AD-relevant deficits in FAD^4T^ mice at baseline. The spatial memory ability and behavioral execution in FAD^4T^ mice were assessed by Morris water maze (MWM) and nest-building test. In the MWM test, AD mice showed obvious learning deficits during the 4 training days (Fig. 7B). On the probe test day, the swimming paths of mice in AD group displayed aimless searching strategy with little improved spatial learning ability, whereas the trajectories of mice after treatment mostly revolved around the platform, especially in the PDA@K group (Fig. 7C). With the same swimming speed, FAD^4T^ mice treated with PDA@K showed improved spatial learning and memory with reduced time to reach the target platform and increased number of platform crossings compared to the 5% glucose-injected groups (Fig. 7D to F). In the nest-building test, PDA@K-treated FAD^4T^ mice achieved a similar score to the WT group, which was much better than the other FAD^4T^ groups (Fig. 7G and H). This also proved that PDA@K could effectively improve the behavior execution ability of FAD^4T^ mice. In addition, we evaluated the therapeutic effect in the Aβ-injected AD model. Behavioral observation including the novel object recognition test and MWM were carried out to examine spatial learning and memory ability. Experimental nesting data showed that Aβ-injected AD mice revealed suppressed interest to novel objects compared with WT mice as determined by the recognition index for novel objects (Fig. S22). After being treated with PDA@K, AD mice showed an obvious preference for novel objects as WT controls. AD mice treated with non-KLVFF-modified PDA performed not so well as PDA@K-treated mice, signifying the importance of the joint action of Aβ recognition peptide and PDA. The same conclusion was obtained in MWM performed at the Aβ-injected AD model (Fig. S23). All the above data confirmed that PDA@K were able to ameliorate the cognitive deficits in AD mice commendably via the joint performance of Aβ recognition by KLVFF and both ROS and metal ion removal by PDA.

### In vivo Aβ clearance, neuroinflammation alleviation, and microenvironment remodeling in FAD^4T^ transgenic mice

**Fig. 8.**
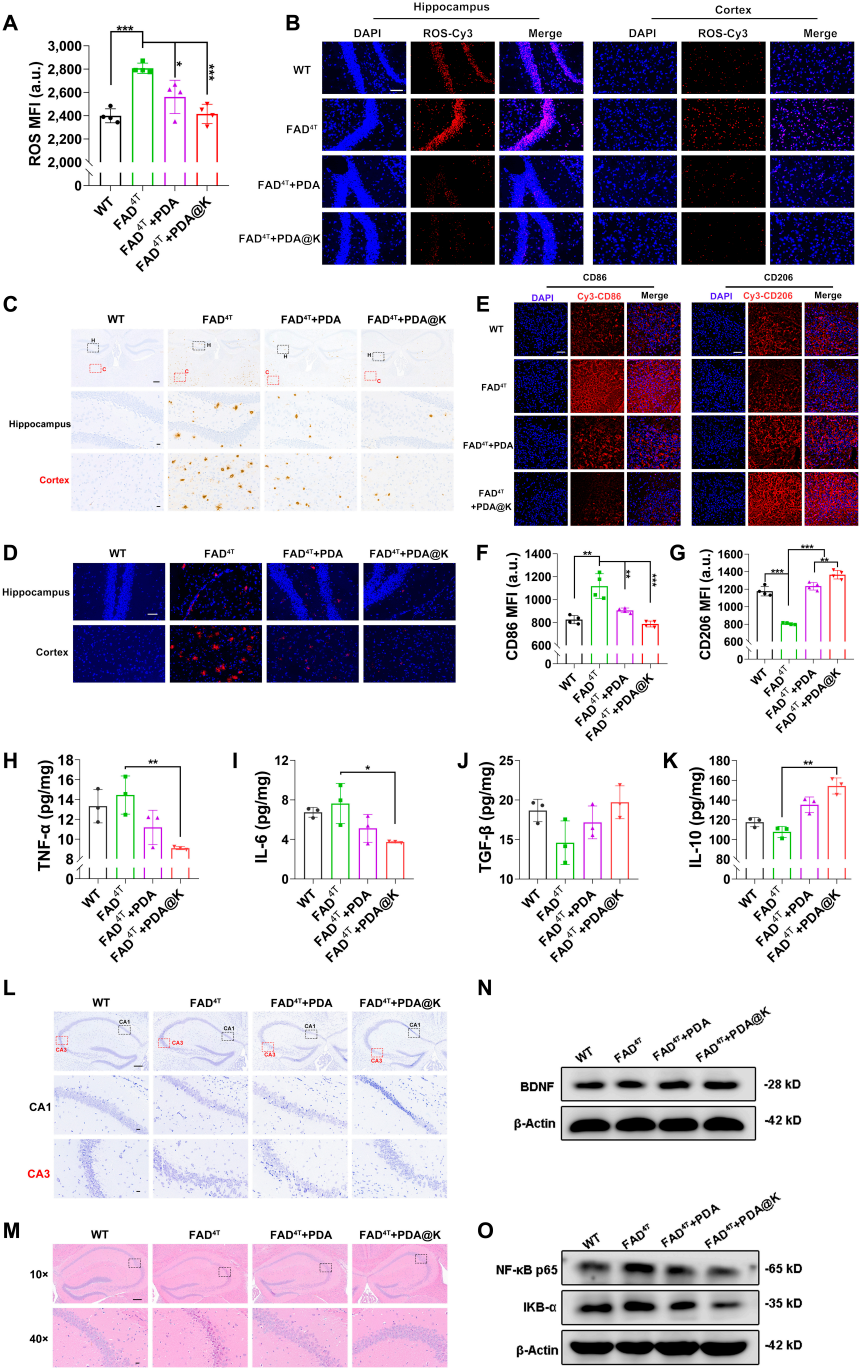
In vivo Aβ clearance, neuroinflammation alleviation, and microenvironment remodeling in FAD^4T^ transgenic mice. (A) Flow cytometry analysis and (B) confocal images for the detection of ROS level in FAD^4T^ transgenic mice brain. Data are presented as mean ± SD, *n* = 4, *P < 0.05, **P < 0.01, ***P < 0.001. Scale bar, 200 μm. (C) Immunohistochemical staining of Aβ plaques in the hippocampus and cortex of FAD^4T^ transgenic mice treated with PDA and PDA@K. First line: scale bar, 200 μm; last 2 lines: scale bar, 20 μm. (D) Immunofluorescence staining of Aβ plaques (red: Aβ; blue: DAPI) in the FAD^4T^ transgenic mice brains. Scale bar, 100 μm. (E) Confocal images of CD86 and CD206 expression analysis of FAD^4T^ mice brain slices. Scale bar, 50 μm. (F) CD86 and (G) CD206 expression analysis of FAD^4T^ mice brain via flow cytometry. Data are presented as mean ± SD, *n* = 4, *P < 0.05, **P < 0.01, ***P < 0.001. (H and I) Expression of pro-inflammatory cytokines TNF-α and IL-6 in the brain after treatment. (J and K) Expression of anti-inflammatory cytokines TGF-β and IL-10 in the brain after treatment. Data are presented as mean ± SD, *n* = 3, *P < 0.05, **P < 0.01. (L) The representative Nissl staining of FAD^4T^ transgenic mice brain after treatment. The black boxes show the hippocampal CA1 area, and the red boxes show the CA3 area. First line: scale bar, 200 μm; last 2 lines: scale bar, 20 μm. (M) HE staining images of FAD^4T^ transgenic mice brain after treatment. The black boxes show the hippocampal CA1 area. First line: 10×, scale bar, 200 μm; last line: 40×, scale bar, 20 μm. (N) Western blotting results of BDNF, (O) NF-κB p65 and IKB-α protein expression. MFI, mean fluorescence intensity.

After behavioral evaluation, mice were sacrificed and tissues were collected for further analysis. Both FCM and fluorescent microscope results showed that the ROS level in the brain of FAD^4T^ mice were significantly higher than that of WT mice, while it was dramatically reduced in those receiving NPs treatment, especially the PDA@K group (Fig. 8A and B). In order to explore the effect of NPs on Aβ deposition, Aβ immunohistochemistry and immunofluorescence staining were adopted to detect the quantity of amyloid plaques. As shown in Fig. 8C and D, abundant Aβ plaques were located in the cortex and hippocampus of FAD^4T^ mice, which was in stark contrast to the WT mice, but the amount and size of Aβ plaques were greatly decreased after treatment. The Aβ plaques in the PDA@K-treated group were almost invisible and close to the level of WT mice, indicating that PDA@K administration was effective in reducing Aβ plaques in brain. Abnormal microglia activation is one of the typical pathological features in AD patients [11,34]. Surprisingly, the M1 marker CD86 expression was downregulated in FAD^4T^ transgenic mice after PDA@K treatment, and M2 marker CD206 expression was upregulated (Fig. 8E to G). In addition, decreased secretion of the pro-inflammatory cytokines (TNF-α and IL-6) and increased expression of the anti-inflammatory cytokines (TGF-β and IL-10) also confirmed the modulation of activated microglia (Fig. 8H to K). These illustrated that PDA@K could reduce excessive ROS, relieve Aβ burden, and stimulate microglial polarization, thereby normalizing the lesion microenvironment in AD brain. Encouraged by the promising pathological features above, we further evaluated neuroprotective performance of NPs morphologically using Nissl staining and hematoxylin and eosin (H&E) staining. The CA1 and CA3 area in the hippocampus were often used for memory study and were prone to lesions [67]. As expected, marked nuclear shrinkage and neuronal damage were observed in FAD^4T^ mice, but not found in WT group. Contrastingly, NPs treatment, especially PDA@K, prominently attenuated the damage of nerve cells (Fig. 8L). The H&E staining of the hippocampus was consistent with the Nissl results (Fig. 8M). We later validated neuroprotective effect at the protein level. Western blot analysis was performed to detect BDNF levels, a protein that proved to be strongly correlated with neuronal repair [68]. The obvious increment of BDNF levels in the PDA@K-treated mice brains indicated that PDA@K treatment could restore BDNF production of AD mice (Fig. 8N and Fig. S24). Furthermore, we found that inflammatory-related proteins such as NF-κB p65 and IKB-α were reduced in the NPs-treated group, especially that treated with PDA@K (Fig. 8O and Fig. S24). Similarly, in the Aβ-injection AD model, obvious ROS removal, microglia polarization, and neuroprotection were also observed after PDA@K treatment (Figs. S25 and S26).

### In vivo transcriptomics analysis

**Fig. 9.**
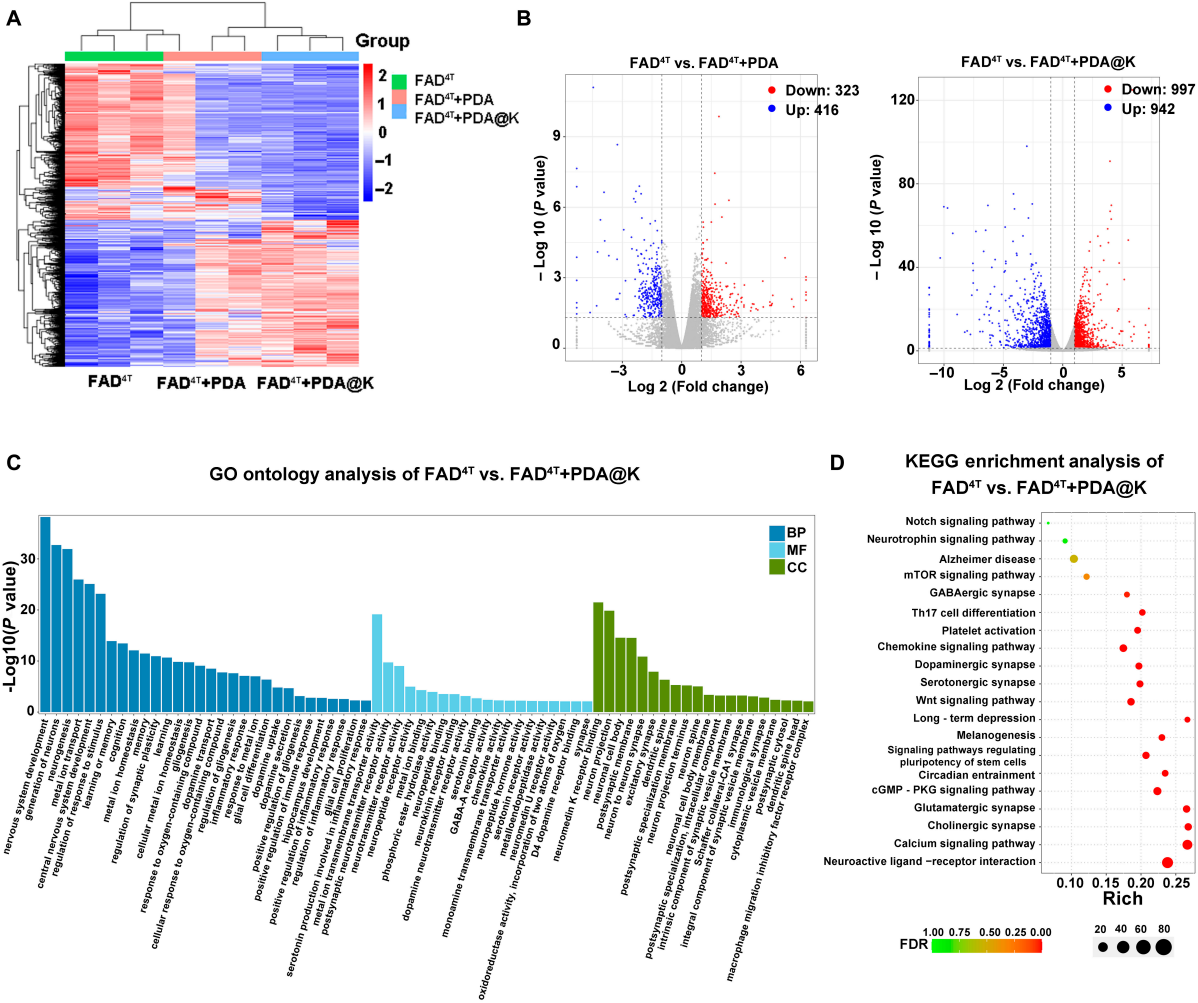
In vivo transcriptomics analysis of FAD^4T^ transgenic mice brains after therapy. Three biological replicates are shown. (A) Heatmap of differentially expressed genes (DEGs) after treated with 5% glucose, PDA, and PDA@K, including the upregulated genes and downregulated genes. (B) Quantitatively analyzed volcano plots of DEGs with NPs treatment compared with 5% glucose treatment (*P* < 0.05, |fold change| ≥ 2). (C) GO enrichment analysis of DEGs exhibiting the involved pathways between PDA@K and 5% glucose-treated FAD^4T^ transgenic mice brains. Biological process, BP; Molecular function, MF; Cell component, CC. (D) KEGG enrichment analysis of the enriched pathways between FAD^4T^+PDA@K and FAD^4T^ group. *n* = 3 mice in each group.

To further understand the therapeutic mechanism of NPs after administration, we evaluated the transcriptomic features of brains of FAD^4T^ mice treated with PDA and PDA@K. The heatmap plot included the differentially expressed genes among the FAD^4T^, FAD^4T^+PDA, and FAD^4T^+PDA@K groups (Fig. 9A). After treatment with PDA@K, a total of 1,939 genes were substantially differentially expressed compared with the FAD^4T^ group, of which 997 transcripts were upregulated (red dots) and 942 genes were downregulated (blue dots) (Fig. 9B). The number of differential genes in the PDA@K-treated group was substantially higher than that in the PDA-treated group. Cluster analysis of expressed genes showed that PDA@K treatment had greatly changed the expression patterns of FAD^4T^ mice brains. Through Gene Ontology (GO) analysis, most of the differentially expressed genes were found to enrich in the categories of the nervous system development, gliogenesis, metal ion homeostasis, inflammatory response, and memory (Fig. 9C). Neuro-related GO terms, for example, nervous system development, generation of neurons, and neurogenesis, were remarkably regulated. Kyoto Encyclopedia of Genes and Genomes (KEGG) enrichment analysis further provided the significantly enriched signaling pathways that are related to AD, such as neuroactive ligand–receptor interaction, circadian entrainment, and dopaminergic synapse (Fig. 9D). Neuroactive ligand–receptor interaction, one of the significantly enriched pathways, has been well documented to be associated with AD. Research had identified its responsibility for regulating the process of neuronal communication, which is crucial for learning and memory [69]. Circadian clock plays a crucial role in neurological diseases. It is reported that regulation of circadian entrainment can control Aβ formation through chronotherapy, thereby alleviating symptoms of AD and inflammatory diseases [70]. Also, the enriched Notch signaling pathway and the mammalian target of rapamycin signaling pathway participated in the pathogenesis and progression of AD and were reported to be compelling targets for AD [71–74]. These data indicated that the therapeutic effect of PDA@K on AD was attributed to the regulation of multiple signaling pathways.

### Systemic toxicity assessment of NPs

Lastly, systemic toxicity of NPs was evaluated. Hemolytic assay revealed that no obvious hemolysis occurred after 24 h of co-incubation (Fig. S27), indicating the overall safety of NPs in vitro. Compared with the WT group, there were no remarkable changes in body weight of the other groups during treatment (Fig. S28). After treatment, major organs including hearts, livers, spleens, lungs, and kidneys from all groups were dissected and stained with H&E. Few differences were noted among these groups (Figs. S29A and S30A). To further assess the systemic response to the NPs, routine blood parameters and chemistry were assessed, including alkaline phosphatase, plasma alanine aminotransferase, aspartate aminotransferase, creatinine, uric acid, and plasma urea, as well as the numbers of leukocytes, neutrophils, lymphocytes, erythrocytes, platelets, and hemoglobin. The liver and kidney functions of mice in each group were in the safe range with no significant difference versus WT mice (Figs. S29B to G and S30B to G). The number of leukocytes, neutrophils, and lymphocytes were all elevated in AD mice, which demonstrate that high levels of inflammation do exist in AD. Surprisingly, all these elevated inflammatory cells returned to the normal range after PDA@K treatment (Fig. S29H to J). This again demonstrated the promising anti-inflammatory effect of PDA@K, and that ROS removal has been proposed as one of the central targets for AD therapy. No other marked changes were found in routine blood parameters (Fig. S29K to M). Taken together, the NPs applied in this study showed good biocompatibility and possessed great potential for clinical applications.

## Discussion

AD is a complex neurodegenerative disorder, and the pathological pathways that govern AD are believed to be mutually reinforced [2]. The sophisticated nature of brain microenvironment and the intricate pathogenesis indicated that single target therapeutics for the rescue of neuronal function is not enough to alleviate the cognitive deficits in AD model mice or AD patients [31,32]. Accordingly, we proposed that multifunctional therapeutics, which can target the pathological BBB, reduce Aβ burden, repolarize microglia, and thus normalize brain microenvironment, would provide a promising alternative to AD treatment. To test this hypothesis, we developed multifunctional biomimetic melanin-like metal ion-chelators PDA@K composed of Aβ recognition peptide KLVFF and synthetic melanin-like nanoparticles PDA to achieve pathological BBB penetration and microenvironment remodeling. As a result, PDA@K had high affinity toward Aβ, were able to hitch a ride on Aβ, and showed enhanced BBB permeation and preference in AD lesion mediated by highly expressed RAGE. PDA@K exhibited strong efficacy in relieving Aβ burden, recovering neuroinflammation, and improving the hippocampal-dependent recognition and spatial memory deficits in AD.

Effective BBB penetration is a big challenge for brain disease therapies. Nanomaterials have been widely explored for enhancing the efficacy of AD therapy due to their nano size effect that increases BBB penetration. However, the pathologically selective BBB crossing strategy still needs to be strengthened as the enhanced penetration only occurred at the lesion site and can be used to achieve precise treatment [75,76]. Inspired by the transport of peripheral Aβ into the brain in AD, the feature of highly expressed RAGE in AD pathology was clearly exploited and combined with Aβ-targeting peptide. By design, our PDA@K-incorporated KLVFF peptide makes it possible to recognize peripheral Aβ, enhancing the delivery of PDA@K across pathological BBB via Aβ hitchhiking mediated by RAGE. In addition to excellent blood stability and effective pathological BBB penetration, we also showed that PDA@K exert high brain accumulation in the AD model, which is a great advancement in AD therapy.

Nanotechnology-based therapy has been monitored under stringent regulatory framework. Clinical translation of nanomedicines requires consideration of multiple factors, including physical and chemical stability, manufacturing, route of administration, distribution, safety, appropriate animal studies, and so on [77]. In our study, the synthetic process of PDA@K is simple and green without high temperature or pressure, which can be adapted to mass production. We demonstrated that our nanomedicine has superior physiological stability and blood longevity that is critical for high accumulation at diseased sites. PDA@K exhibited excellent biocompatibility and did not cause renal or hepatic responses or adverse effects, suggesting that there is an effective clearance of by-products from the body. Our previous study has shown that nanomedicine could be eliminated from brain, resulting in good biosafety [78]. The simple and controllable synthetic route and excellent safety hold promise for clinical translation. In addition, the intrinsically effective functions of bioactive PDA-metal chelation and ROS scavenging were utilized for AD therapy, which simplifies the entire drug delivery system. Our work inspired the possibility of exploring functional nanomaterials that are directly used as anti-AD drugs rather than just acting as nanocarriers. In future studies, the functional PDA@K could be applied to carry multiple clinical drugs for AD treatment.

The cascade reactions triggered by the exacerbation of inflammatory microenvironment in AD brain are gaining attention. The overactivation of microglia in AD lesions affects the degradation of toxic proteins, and worse still, active microglia release inflammatory factors and promote ROS generation, which accelerates neuronal necrosis [11,62]. The increasingly prominent role of microglia dysfunction in AD allowed the restoration of microglial homeostasis to be taken into consideration of therapeutic strategies. In summary, based on complex etiologies in AD development, the multifunctional melanin-like metal ion chelators were reported here to reduce Aβ burden and normalize inflammatory microenvironment synergistically through its intrinsic capacity. The KLVFF peptide could not only enhance pathological BBB permeation but also capture disaggregated Aβ to promote microglial uptake of extracellular Aβ. The bioactive PDA chelated metal ions to accelerate Aβ depolymerization, which, together with their powerful ROS scavenging ability, reduced neuroinflammation and normalized brain microenvironment. On the one hand, they reduced the Aβ-related neurotoxicity, and on the other hand, the normalized conditions accelerated microglia to degrade Aβ. These positive pathophysiological effects contributed to the restoration of cognitive performance of PDA@K-treated FAD^4T^ transgenic mice. Collectively, our study provided convincing evidence for considering metal dyshomeostasis and inflammatory microenvironment as therapeutic targets of AD, which could be considered as a novel multi-pronged strategy in the exploration of AD treatments.

## Materials and Methods

### Materials

Dopamine hydrochloride and ThT were purchased from Sigma-Aldrich Co., LLC (USA). KLVFF peptide chain was synthesized by Sangon Biotech Co., Ltd (Shanghai, China). NH_2_-PEG-CH_2_CH_2_COOH, NH_2_-PEG-SH and Cy5 NHS ester were purchased from Dalian Meilun Biotech Co., Ltd. (Dalian, China). 2′,7′-Dichlorodihydrofluorescein diacetate (DCFH-DA) was obtained from Bide Pharmatech Co., Ltd. (Shanghai, China). Complete RPMI-1640, Dulbecco’s modified Eagle’s medium (DMEM), trypsin-EDTA solutions, and fetal bovine serum (FBS) were purchased from Gibco (USA). Amyloid-β (1–42) peptide (Aβ_42_) and FITC-amyloid-β (1–42) peptide (FITC-Aβ_42_) were purchased from GL Biochem Ltd. (Shanghai, China). Anti-beta amyloid antibody (ab201060) and anti-IKB alpha antibody (ab32518) were purchased from Abcam (UK). NF-κB p65 antibody was obtained from Cell Signaling Technology, Inc. (USA). BDNF antibody was obtained from ABclonal Technology Co., Ltd. (Wuhan, China). Anti-CD206 and -CD86 antibodies were obtained from Proteintech Group, Inc. (Wuhan, China). Enzyme-linked immunosorbent assay (ELISA) kits (TNF-α, IL-6, IL-10, and TGF-β) were purchased from BD Pharmingen (USA). All the chemicals used were analytical or reagent grade.

### Cell lines and animals

The bEnd.3, BV2, and PC-12 cell lines were obtained from the Chinese Academy of Sciences Cell Bank (Shanghai, China). Cells were cultured in complete RPMI-1640 or DMEM containing 10% FBS and 100 U/ml penicillin–streptomycin and retained in an incubator at 37 °C and 5% CO_2_. BALB/c and C57BL/6 mice were purchased from Chengdu Dashuo Lab. Animal Co., Ltd. (Chengdu, China). FAD^4T^ [B6/JGpt-Tg(Thy-APP/Thy-PSEN1)5/Gpt] mice were provided by the Gempharmatech Co., Ltd (Jiangsu, China). All animals were maintained under standard housing conditions. All animal experiments were carried out under the guidelines approved by the experimental animal management committee of Sichuan University.

### Intracellular ROS scavenging

PC-12 cells (1× 10^5^ cells per well) were planted in 12-well plates and incubated with 100 μg/ml PDA or PDA@K for 24 h after grown to 70% to 80% confluence. The positive controls were treated with TBH for 6 h. After that, the medium was replaced with FBS-free medium containing 10 μmol/l DCFH-DA in each well and incubated for 30 min at 37 °C. Then, cells were harvested and washed with PBS 3 times to fully remove excessive DCFH-DA. The FI was analyzed using a flow cytometer (Agilent NovoCyte, USA).

For fluorescent microscope analysis, PC-12 cells were planted into the coverslip in 12-well plates and treated the same as the FCM assay. After receiving DCFH-DA treatment, the cells were washed with PBS, fixed with 4% paraformaldehyde, and stained by 4', 6-diamidino-2-phenylindole (DAPI) (1 μg/ml). The DCFH-DA FI was imaged by a fluorescence microscope (Leica, DMi8, Germany).

### ThT assay

The concentrated ThT solution (2 mmol/l) and Aβ_42_ (1 mg/ml) dissolved in PBS were prepared in advance. For each measurement, the prepared Aβ_42_ solutions with or without NPs and metal ions were co-incubated with ThT solutions in glycine/NaOH (90 mmol/l, pH 8.5) to the final concentration of 20 μmol/l for Aβ_42_ and 20 μmol/l for ThT. The molar ratio of Aβ_42_/samples was 1/2. The mixtures were added to 96-well plates at 200 μl per well. After 24 h incubation with shaking at 60 rpm at 37 °C, the ThT FI was measured with a microplate reader (Ex: 440 nm, Em: 485 nm).

### Aβ fibril inhibition and disaggregation experiments

The inhibition and disaggregation of Aβ experiments were performed to study the effect of NPs on metal-induced Aβ fibril aggregation. Aβ_42_ monomers (10 μmol/l) were incubated with different concentrations of Zn^2+^ or Cu^2+^ for testing the effect of metal ions on Aβ aggregation. In the inhibition study, Zn^2+^ or Cu^2+^ metal ions were added into Aβ_42_ solution (10 μmol/l) at the final concentration of 400 ng/ml. PDA and PDA@K were introduced respectively at a concentration of 0.4 mg/ml and incubated for 24 h at 37 °C. In the disaggregation experiment, Aβ_42_ was incubated for 24 h in PBS firstly at 37 °C to form Aβ fibers (Aβ_42(24h)_). Then, PDA and PDA@K (0.4 mg/ml) were introduced into pre-incubated Aβ_42(24h)_ solution (10 μmol/l), respectively. The samples were cultured for 24 h at 37 °C. The effects of Aβ_42_ fibril inhibition and disaggregation were confirmed by ThT fluorescence assay. In order to verify whether serum affected the metal chelating ability of NPs and thus hindered their inhibitory effect on Aβ_42_ aggregation, PDA and PDA@K were incubated with mouse serum for 24 h at 37 °C and recycled. After that, the serum-incubated NPs (0.4 mg/ml) were mixed with Aβ_42_ solution (10 μmol/l) with or without metal ions for 24 h at 37 °C. The degree of Aβ aggregation was observed by ThT assay.

### In vitro pathological BBB crossing assay

In the BBB mimetic model, bEnd.3 cells were seeded in the upper chambers [79] (12 well, pore size: 3 μm, Corning, USA). The culture medium was replaced every day and the TEER values of the bEnd.3 layer were recorded every 2 days (Millicell-ERS, Millipore, USA). After the TEER value reached 180 Ω/cm^2^, 8×10^4^ BV2 cells were seeded per well in the lower chamber. Two days later, new medium containing Aβ (5 μmol/l) was added into corresponding wells for 24 h. Then, the medium was removed and washed with PBS twice. RB-labeled NPs (100 μg/ml) with or without low concentration Aβ (4.5 nM) were added into the upper inserts. After 4 h of incubation, the FI of each lower chamber supernatant was detected via a microplate reader. The mean FI of RB in BV2 cells was detected using FCM and a confocal microscope. 3D confocal microscopic imaging was used to observe the penetration of NPs in the bEnd.3 layer.

### In vivo distribution of NPs

The Aβ-injection AD model was established according to the published methods [80]. Aβ_42_ (1 mg/ml) was pre-incubated in artificial cerebrospinal fluid at 37 °C for 7 days. Mice were anesthetized and fixed on a brain stereotactic fixation device for surgery. Pre-incubated Aβ_42_ (5 μl) was slowly injected into the right hippocampal region. After 2 weeks of recovery, AD mice were randomly grouped and sham-operated mice were used as healthy control. One hundred microliters of Cy5-labeled NPs (PDA and PDA@K, 30 mg/kg) were administrated intravenously at the same concentration of Cy5. At 2, 4, 8, 12, and 24 h post-administration, mice were anesthetized and imaged using the Lumina III Imaging System (PerkinElmer, USA). At 24 h, the mice were sacrificed, and their major organs and brains were separated for ex vivo fluorescence imaging. After that, the organs and brains were fixed with 4% paraformaldehyde, dehydrated in sucrose, embedded by tissue-tek O.C.T compound (Sakura Finetek, USA), and then sectioned (Leica CM1950, Germany). All the slices were stained with DAPI (1 μg/ml) and observed under a confocal microscope.

### MWM experiment

Five-month-old FAD^4T^ transgenic mice were randomly divided into 3 groups, 5% Glucose, PDA, and PDA@K (10 mice per group). The same number of WT C57BL/6 mice were used as healthy control group. Five percent glucose and NPs (PDA and PDA@K, 30 mg/kg) were intravenously administered once every 3 days for 7 doses, respectively. The body weights were recorded every 3 days for toxicity assessment. After treatment, all mice were trained and tested in a water maze with a diameter of 80 cm to evaluate spatial learning and memory ability. In detail, the maze was filled with water and replaced daily. The water temperature was maintained at 20 ± 1 °C. A circular platform (10 cm in diameter) was immobilized to 1 cm under the water surface during the training period and a camera detection device was used for tracing movement. In the first 4 days, all mice were trained to swim to find the platform from 3 diverse positions around the border of the maze. If the mice found the hidden platform within 60 s successfully, they were allowed to stay on the platform for 10 s. If they failed, the mice were guided to the platform and placed there for 10 s. After training for 4 days, the platform was removed and the spatial probe test was carried out. Every trained mouse was allowed to swim freely for 60 s. The latency to reach the platform, the time spent in the target quadrant where the platform used to be, and the number of initial platform crossings were recorded for spatial memory evolution.

### Nest-building test

The mice were housed one mouse per cage and placed in the test lab for adaption to 48 h. Then, 10 pieces of thin paper (5 × 5 cm) were introduced in the cage to provide conditions for mice to build nests. Twenty-four hours later, the nests were photographed and scored to assess nest-building behavior. The nest scores were based on the following criteria: (a) no obvious nest sites, and the paper was not torn; (b) the paper was not torn obviously, but the nest site was identifiable; (c) partially torn paper with identifiable nest sites; and (d) most of the paper towels were bitten with identifiable nest sites. All results were scored blindly.

### Mechanism experiments in vivo

After behavioral experiment, mice blood was collected before execution for routine blood parameter and chemistry analysis. The mice brains were isolated and the cross-sections of the hippocampus of some brains were selected for immunohistochemistry and immunofluorescence staining, ROS staining, Nissl, and H&E staining, respectively. Some mice brain tissues were ground to collect single cells for FCM analysis of CD86, CD206 expression, and the level of ROS detection. Transcriptomics analysis of some mice brains was performed by the BioNovoGene (Suzhou, China). The hippocampal tissues in some brains were homogenized by a high-speed tissue grinder (Servicebio, Wuhan, China) to perform Western blot and ELISA. The major organs including hearts, livers, spleens, lungs, and kidneys were sectioned and stained with Nissl and H&E to check the cumulative toxicity of NPs in vivo.

### Statistical analysis

Statistical analysis was obtained with GraphPad Prism (version 8, CA). Data were expressed as mean ± SD, and analyzed by 2-sided unpaired Student’s *t* test with significance defined as *P < 0.05, **P < 0.01, ***P < 0.001, compared with control. Data were representative of 3 dependent experiments.

## Data Availability

All data are available in the main text or the Supplementary Materials. Additional data related to this paper may be requested from the authors.

## Supplementary Materials

Supplementary 1 Fig. S1. MALDI-TOF-MS analyses of amine-terminated polyethylene glycol peptide (NH_2_-PEG8-_(Ac)_KLVFF). Fig. S2. ^1^H nuclear magnetic resonance (^1^H NMR, 400 MHz) spectra of NH_2_-PEG8-_(Ac)_KLVFF. Fig. S3. UV spectra of the KLVFF peptide at 250 to 260 nm in different concentrations. Fig. S4. Zeta potentials of PDA before and after capturing Cu^2+^ and Zn^2+^. Fig. S5. Schematic diagram of the nitroblue tetrazolium (NBT) method for testing O_2_•− scavenging efficiency by NPs. Fig. S6. The calculated scavenging efficiency of •OH after incubation with different concentrations of PDA. Fig. S7. UV spectra of H_2_O_2_ in different concentrations and the standard curve of H_2_O_2_ based on the UV values at 240 nm. Fig. S8. Cell viability of PC-12 cells after incubation with different concentrations of PDA@K for 24 h and 48 h measured by MTT assay, respectively. Fig. S9. Representative flow cytometry plots showing fluorescence levels of cells in different experimental conditions. Fig. S10. Changes in cell morphology after 2 h of TBH treatment in the presence and absence of NPs. Fig. S11. ThT assay for evaluation of the effect of different concentrations of metal ions on Aβ aggregation. Fig. S12. The effect of serum and the inhibitory effect on Aβ aggregation of NPs in the presence of metal ions. Fig. S13. Fluorescence intensity curves of different concentrations of Rhodamine B 5-isothiocyanate (RBITC). Fig. S14. Validation of microglia polarization toward the M1 type under different levels of oxidative stress. Fig. S15. Fluorescence intensity of the remaining Aβ in the supernatant after co-incubation of different concentrations of PDA@K with Aβ solutions. Fig. S16. (A) Western blotting results of RAGE expression in bEnd.3 cells with or without Aβ (10 μM) incubation for 24 h. Fig. S17. Fluorescence intensity of BV2 cells in the lower chamber measured by FCM for BBB permeability evaluation. Fig. S18. Fluorescent images of BV2 cells in the lower chamber of BBB for BBB permeability evaluation. Fig. S19. Fluorescence intensity curves of different concentrations of Cy5. Fig. S20. Fluorescent distribution of PDA and PDA@K in the frozen sections of major organs. Fig. S21. The relative serum concentration of Aβ_42_ of WT, 2 weeks after the Aβ-injected model, and FAD^4T^ transgenic mice measured by ELISA. Fig. S22. The novel object recognition test of AD mice (Aβ-injected mice) after therapy. Fig. S23. Morris water maze (MWM) test of AD mice (Aβ-injected mice) after therapy. Fig. S24. The corresponding semi-quantitative results of (A) BDNF (*n* = 4), (B) NF-κB p65 (*n* = 3), and (C) IKB-α protein expression (*n* = 3) in FAD^4T^ mice brain after therapy. Fig. S25. (A) Flow cytometry analysis and (B) confocal images for the detection of ROS level in AD mice brain (Aβ-injected mice). Fig. S26. (A) CD206 and (B) CD86 expression analysis of AD mice brain (Aβ-injected mice) via flow cytometry after therapy. Fig. S27. Hemolysis test of NPs in different concentrations. Fig. S28. Body weight changes of FAD^4T^ transgenic mice and Aβ-injected mice during treatment. Fig. S29. Systemic toxicity assessment of NPs in FAD^4T^ transgenic mice after treatment. Fig. S30. Systemic toxicity assessment of NPs in Aβ-injected AD mice after treatment.
