# Supplementary material for: Pathological BBB Crossing Melanin-Like Nanoparticles as Metal-Ion Chelators and Neuroinflammation Regulators against Alzheimer’s Disease
Source: Research (Wash D C). 2023 Jun 23;6:0180. doi: 10.34133/research.0180 (PMC10289297; doi:10.34133/research.0180)
Supplement: Supplementary 1 — Fig. S1. MALDI-TOF-MS analyses of amine-terminated polyethylene glycol peptide (NH2-PEG8-(Ac)KLVFF). Fig. S2. 1H nuclear magnetic resonance (1H NMR, 400 MHz) spectra of NH2-PEG8-(Ac)KLVFF. Fig. S3. UV spectra of the KLVFF peptide at 250 to 260 nm in different concentrations. Fig. S4. Zeta potentials of PDA before and after capturing Cu2+ and Zn2+. Fig. S5. Schematic diagram of the nitroblue tetrazolium (NBT) method for testing O2•− scavenging efficiency by NPs. Fig. S6. The calculated scavenging efficiency of •OH after incubation with different concentrations of PDA. Fig. S7. UV spectra of H2O2 in different concentrations and the standard curve of H2O2 based on the UV values at 240 nm. Fig. S8. Cell viability of PC-12 cells after incubation with different concentrations of PDA@K for 24 h and 48 h measured by MTT assay, respectively. Fig. S9. Representative flow cytometry plots showing fluorescence levels of cells in different experimental conditions. Fig. S10. Changes in cell morphology after 2 h of TBH treatment in the presence and absence of NPs. Fig. S11. ThT assay for evaluation of the effect of different concentrations of metal ions on Aβ aggregation. Fig. S12. The effect of serum and the inhibitory effect on Aβ aggregation of NPs in the presence of metal ions. Fig. S13. Fluorescence intensity curves of different concentrations of Rhodamine B 5-isothiocyanate (RBITC). Fig. S14. Validation of microglia polarization toward the M1 type under different levels of oxidative stress. Fig. S15. Fluorescence intensity of the remaining Aβ in the supernatant after co-incubation of different concentrations of PDA@K with Aβ solutions. Fig. S16. (A) Western blotting results of RAGE expression in bEnd.3 cells with or without Aβ (10 μM) incubation for 24 h. Fig. S17. Fluorescence intensity of BV2 cells in the lower chamber measured by FCM for BBB permeability evaluation. Fig. S18. Fluorescent images of BV2 cells in the lower chamber of BBB for BBB permeability evaluation. Fig. [file research.0180.f1.docx]

Supplementary Materials

**Pathological BBB Crossing Melanin-like Nanoparticles as Metal-ion Chelator and Neuroinflammation Regulator against Alzheimer’s Disease**

Qianqian Huang, Chaoqing Jiang, Xue Xia, Yufan Wang, Chenxing Yan, Xiaorong Wang, Ting Lei, Xiaotong Yang, Wenqin Yang, Guo Cheng*, and Huile Gao*

*Corresponding author. Email: [gaohuile@scu.edu.cn](mailto:gaohuile@scu.edu.cn); [gcheng@scu.edu.cn](mailto:gcheng@scu.edu.cn).

**This PDF file includes:**

Supplementary Text

Figs. S1 to S30

Supplementary Text

**Preparation and characterization of NPs**

**Preparation of PDA**: The PDA were synthesized via dopamine oxidation and self-polymerization in Tris buffer at specific pH. Briefly, 1 mg/mL dopamine hydrochloride was dissolved in 20 mM Tris at pH 11.5 and allowed to stir (400 rpm) for 1 h at 37 °C. The synthesized PDA were isolated via centrifugation at 12000 rpm for 15 min and washed three times with deionized water. The obtained PDA were lyophilized and weighed for quantitation.

**Preparation of PDA@K**: Amine-terminated polyethylene glycol peptide (NH_2_-PEG-_(Ac)_KLVFF) was modified on the surface of PDA (PDA@K) through Schiff base and/or Michael addition reactions. In detail, PDA were mixed with NH_2_-PEG-_(Ac)_KLVFF and NH_2_-mPEG at a mass ratio of (2:2:1) in alkaline buffer solution (pH = 8.5) under vigorous stirring for 24 h. After that, the solution was purified by centrifugation (12000 rpm, 15 min) and washed three times with deionized water.

**Preparation of Rhodamine B (RB)-labeled NPs**: RB-labeled PDA were obtained via π-π conjugation between RB and PDA. For the synthesis of RB-labeled PDA, RB stock solution (10 mg/mL in DMSO) was added to the as-synthesized PDA colloid at a final RB concentration of 20 μg/mL. The mixture was allowed to react at 25 °C for over 24 h under magnetic stirring. The mixture was washed thrice after incubation by repeated centrifugation (12000 rpm, 15 min).

**Preparation of Cy5-labeled NPs**: SH-PEG-Cy5 was synthesized in advance via the reaction between SH-PEG-NH_2_ and Cy5-NHS. To be specific, Cy5-NHS stock solution dissolved in DMSO (10 mg/mL) was mixed with SH-PEG-NH_2_ in a molar ratio of 1.5:1 at a final concentration of 2 mg/mL. Add 2 equivalents of triethylamine and stirred for 24 h at 37 ℃ under light-proof conditions. The reaction solution was dialyzed with DMSO and water for 24 h, respectively. Then the samples were lyophilized and stored at -20℃ for future use. For the synthesis of Cy5-labeled PDA, SH-PEG-Cy5 synthesized before were dissolved in DMSO to form a stock solution at 10 mg/mL. SH-PEG-Cy5 stock solution was mixed with PDA solution (0.4 mg/mL) at a volume ratio of 1: 100 in alkaline condition (pH = 8.5) and reacted over 24 h at 25 °C under magnetic stirring. Then, the product was collected and purified three times using deionized water by centrifugation (12000 rpm, 15 min).

**Characterization of materials and NPs**

The synthesis of the amine-terminated polyethylene glycol peptide (NH_2_-PEG-_(Ac)_KLVFF) was confirmed by matrix-assisted laser desorption/ionization-time-of-flight mass spectrum (MALDI-TOF-MS, Shimadzu, Japan) and proton nuclear magnetic resonance (^1^H NMR) spectrum. The linkage of peptide chain on NPs was verified by UV absorption and Fourier infrared spectroscopy (IR), zeta potential respectively. The concentration of unreacted peptide in the supernatant was determined using UV spectrophotometer. Dynamic light scattering (DLS, Brookhaven) and transmission electronic microscopy (TEM, H-600, Hitachi, Japan) were used for monitoring NPs size, morphology, and zeta potential.

**Scavenging of •OH**

•OH scavenging efficiency was assessed by measuring the FI of existing 2-hydroxyterephthalic acid. Terephthalic acid (TA) (0.5 mM) and H_2_O_2_ (10 mM) were added in different concentrations of PDA (0, 12.5, 25, 50, 75, 100, 200 μg/mL) and shook at 60 rpm at 37℃ for 12 h. TA here was used as a nonfluorescent compound could capture •OH to produce 2-hydroxyterephthalic acid. After that, the FI (Ex: 320 nm, Em: 425 nm) was measured via fluorospectrophotometer for •OH clearance assessment.

**Scavenging of O_2_·^−^**

O_2_**·**^−^ scavenging by PDA was analyzed using a superoxide dismutase (SOD) determination kit (Beyotime). For details, PDA solution (20 µL) were added to 200 µL of a 2-(4-Iodophenyl)-3-(4-nitrophenyl)-5-(2,4-disulfophenyl)-2H-tetrazolium monosodium salt working solution and thorough mixed. The final concentrations PDA were 0, 12.5, 25, 50, 75, 100, 200 μg/mL, respectively. After 30 min incubation at 37 °C, the absorbances at 560 nm were measured to calculate the scavenging efficiency of O_2_**·**^−^.

**Clearance of H_2_O_2_**

For H_2_O_2_ scavenge assay, different concentrations of H_2_O_2_ solutions were prepared and their UV absorptions at 240 nm were measured to plot the standard curve. Then, H_2_O_2_ (10 mM) was mixed with different concentrations of PDA (0, 12.5, 25, 50, 75, 100, 200 μg/mL) for 6 h at 37 ℃, individually. The same concentrations of PDA without H_2_O_2_ were used as controls. The UV absorptions of above solutions were measured at 240 nm to calculate H_2_O_2_ scavenging efficiency. All tests were performed in triplicate (n = 3)

**Cellular uptake and retention**

Cells were seeded into 12-well plate at a density of 8 × 10^5^ per well. After 24 h, the culture medium was removed and washed with PBS twice. RB-labeled NPs diluted in culture medium (50, 100, 200 μg/mL) were added into the plate and incubated for 4 h. Then, the cells were washed with PBS three times and harvested for cellular uptake testing via FCM.

**Internalization analysis of Aβ**

When BV2 cells seeded into 12-well plates (8 × 10^5^ cells per well) and grew to 80%, fresh medium containing 2 μmol/L Aβ_42_-FITC and 100 μg/mL RB-labeled NPs (PDA, PDA@K) were added into the corresponding wells. After 4 h incubation, the cells were harvested and washed with PBS three times. The FI of FITC was analyzed using FCM.

**Fluorescence co-localization assay**

For co-localization assay, BV2 cells were seeded into the coverslip in 12-well plates (8 × 10^5^ cells per well). The same concentration of Aβ_42_-FITC and RB-labeled NPs were added into the corresponding wells after cells grown to 80% confluent. 4 h later, cells were washed three times with PBS, fixed by 4% paraformaldehyde and stained with DAPI (1 μg/mL) for 5 min. Co-localization was observed with laser scanning confocal microscopy (Eclipse Ti, Nikon, Japan).

**Peripheral Aβ targeting test**

An Aβ42-FITC stock solution with a concentration of 1mg/mL was prepared in advance. The stock solution was diluted in a gradient to a final concentration of 4.5 nM to ensure the accuracy. After that, the prepared Aβ_42_ solution was incubated with different concentrations of PDA@K (0, 6.25, 12.5, 25, 50, 100 μg/mL) for 12 h at 37 ℃ under light-proof conditions, respectively. All samples were centrifuged at 12,000 rpm for 15 min to collect supernatant. The sample without PDA@K was labeled as Control. The remaining fluorescence intensities of the supernatant were measured with a microplate reader. All tests were performed in triplicate (n = 3).

**Novel object recognition (NOR) test**

The NOR test was divided into three parts: the habituation stage, training stage, and the testing stage (short-time and long-time testing). During the habituation stage, mouse was placed in the middle of the open field without any object inside and let it explore the field for 5 min. After 24 h, the mouse was placed in the same box, which had been placed with same objects in two opposite corners in advance. Mice were allowed to explore the objects for 10 min. The short-time test begun 2 h after the training stage. For short-time test (test 1), one of the objects was replaced with a novel one, and mice were allowed to explore the objects for 5 min. After 24 h, another object was replaced with a novel one, and mice were allowed to explore objects for 5 min for long-time test (test 2). All the trajectories of mice were recorded during the whole process. In the training stage, the time for mice to explore the one object was defined as T1, and the time to explore another object was T2. The recognition index (RI) was calculated with the formula: RI = T1/(T1+T2) ×1 00%. In test 1 and test 2, the time to explore the old object was T1, and the time to explore the novel object was T2. RI = T2/(T1+T2) × 100%.

Supplementary Figures


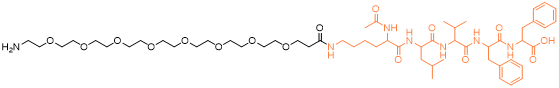


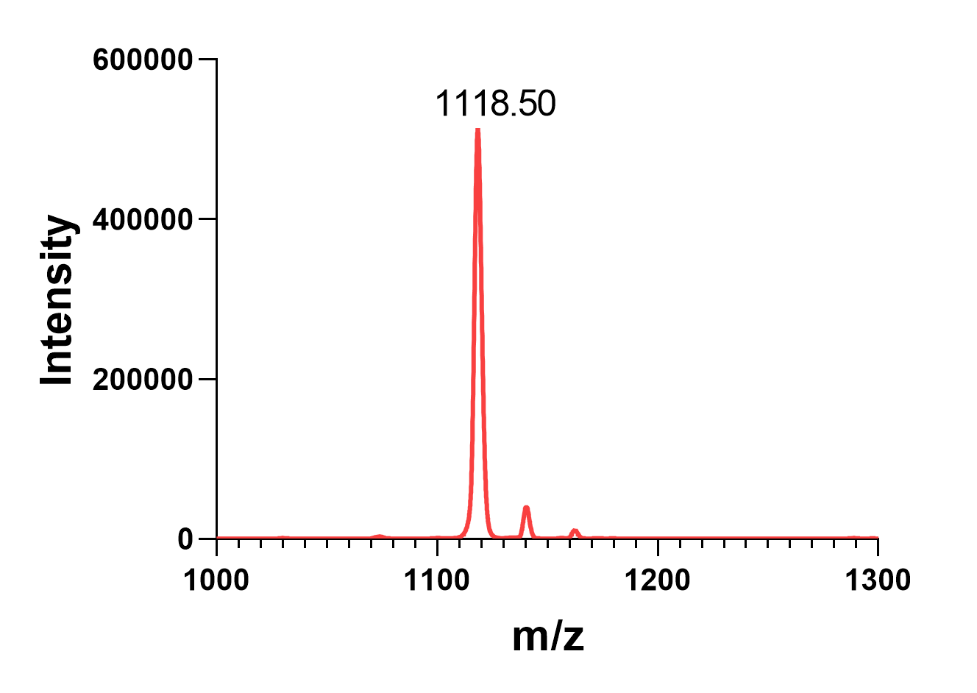


***Fig. S1.*** *MALDI-TOF-MS analyses of amine-terminated polyethylene glycol peptide (NH_2_-PEG8-_(Ac)_KLVFF). The peaks at m/z 1118.5 belonged to NH_2_-PEG8-_(Ac)_KLVFF.*

**
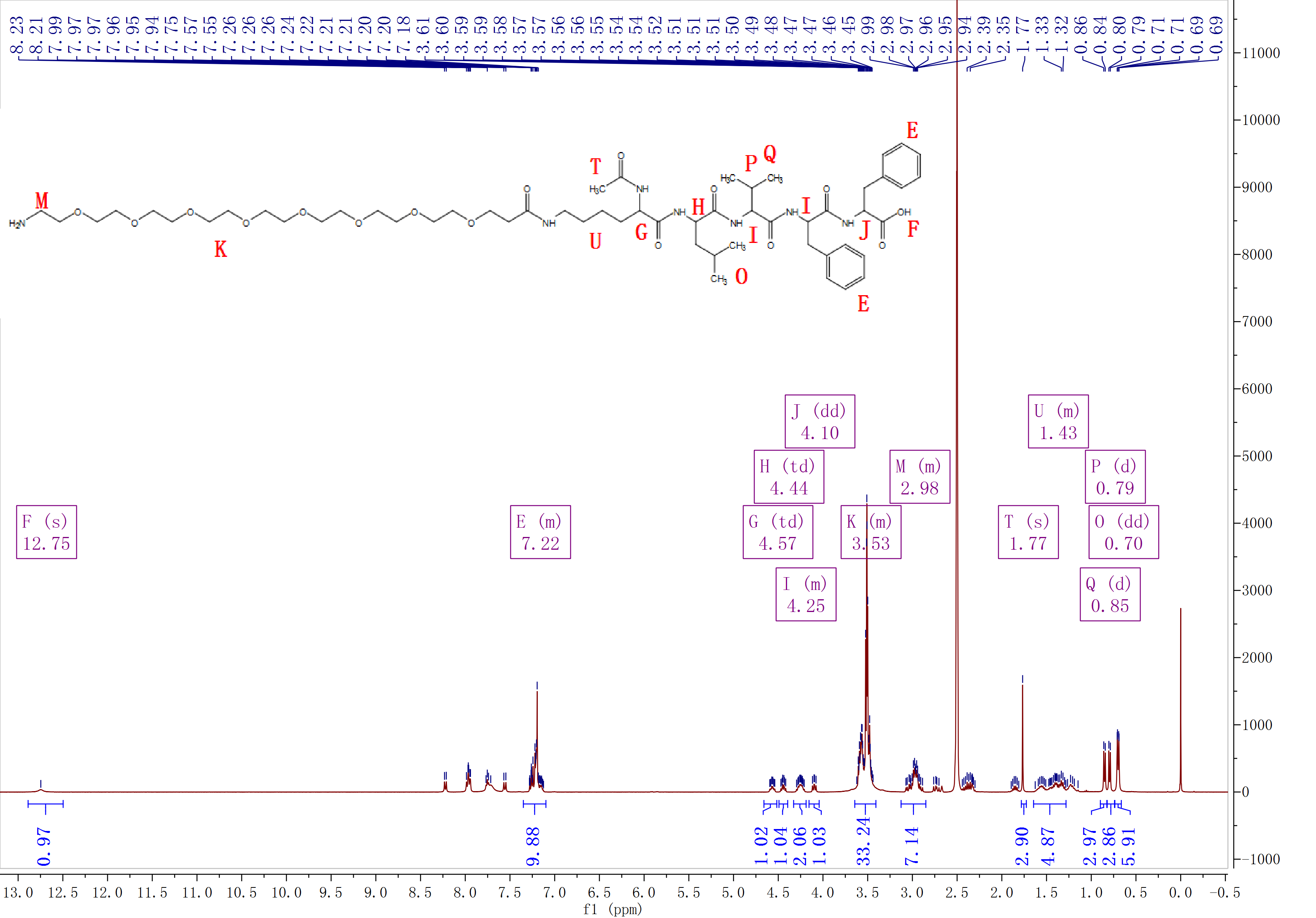
**

***Fig. S2.*** *^1^H nuclear magnetic resonance (^1^H NMR, 400 MHz) spectra of NH_2_-PEG8-_(Ac)_KLVFF.*

**
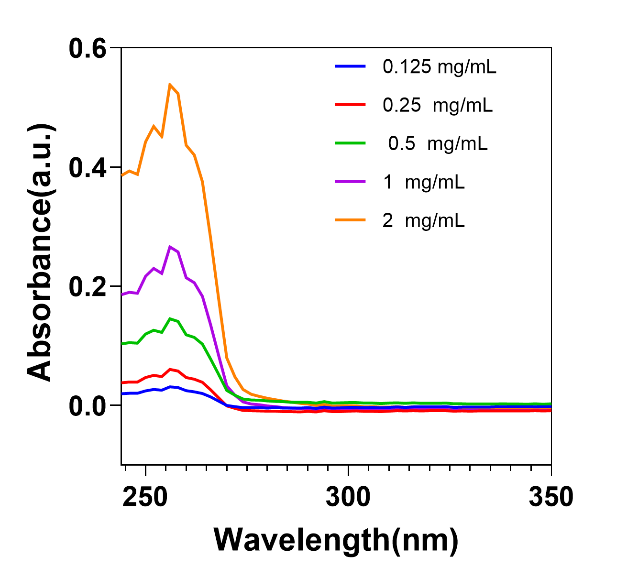
**

***Fig. S3.*** *UV spectra of KLVFF peptide at 250-260 nm in different concentrations.*

**
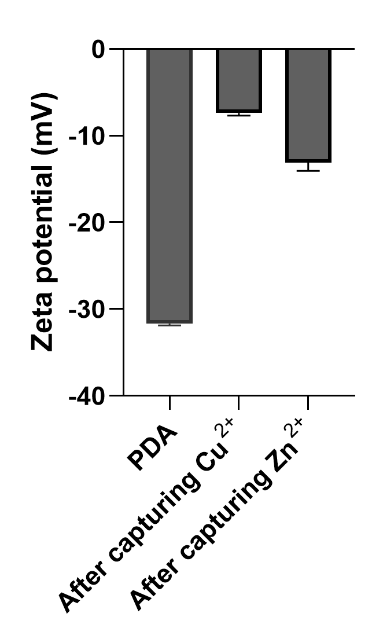
**

***Fig. S4.*** *Zeta-potentials of PDA before and after capturing Cu^2+^, Zn^2+^. Data presented as mean ± SD, n = 3.*

**
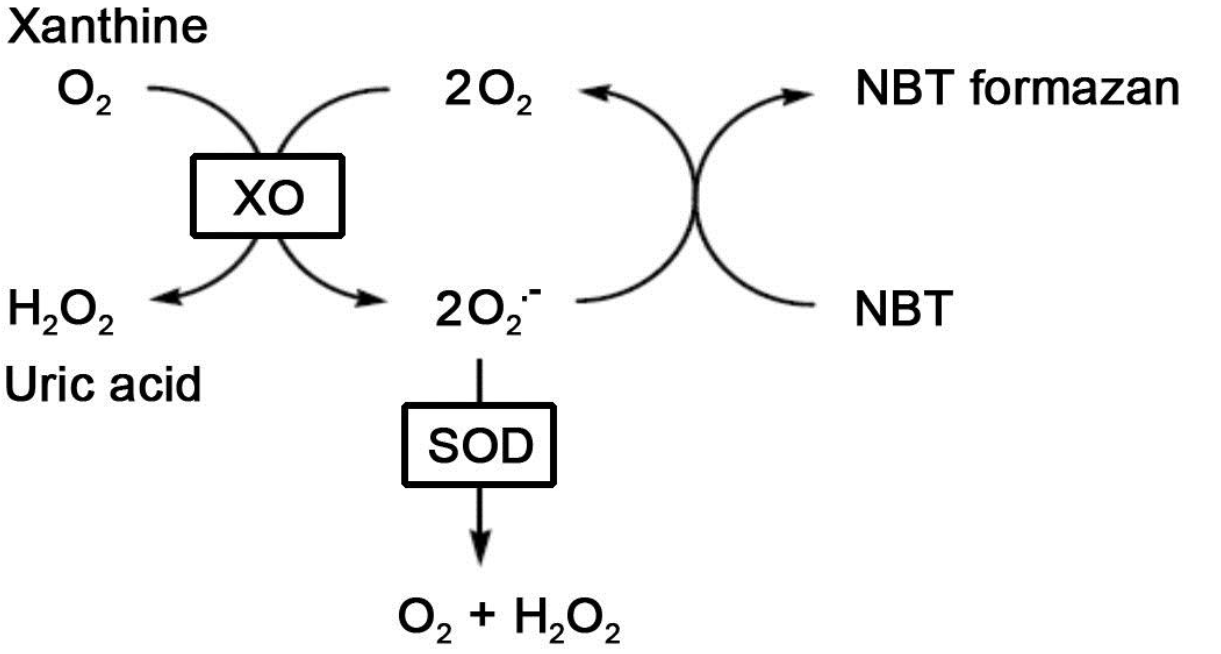
**

***Fig. S5.*** *Schematic diagram of the nitroblue tetrazolium (NBT) method for testing O_2_•^−^ scavenging efficiency by NPs. The detailed experimental procedures are described in the Supplementary text section.*

**
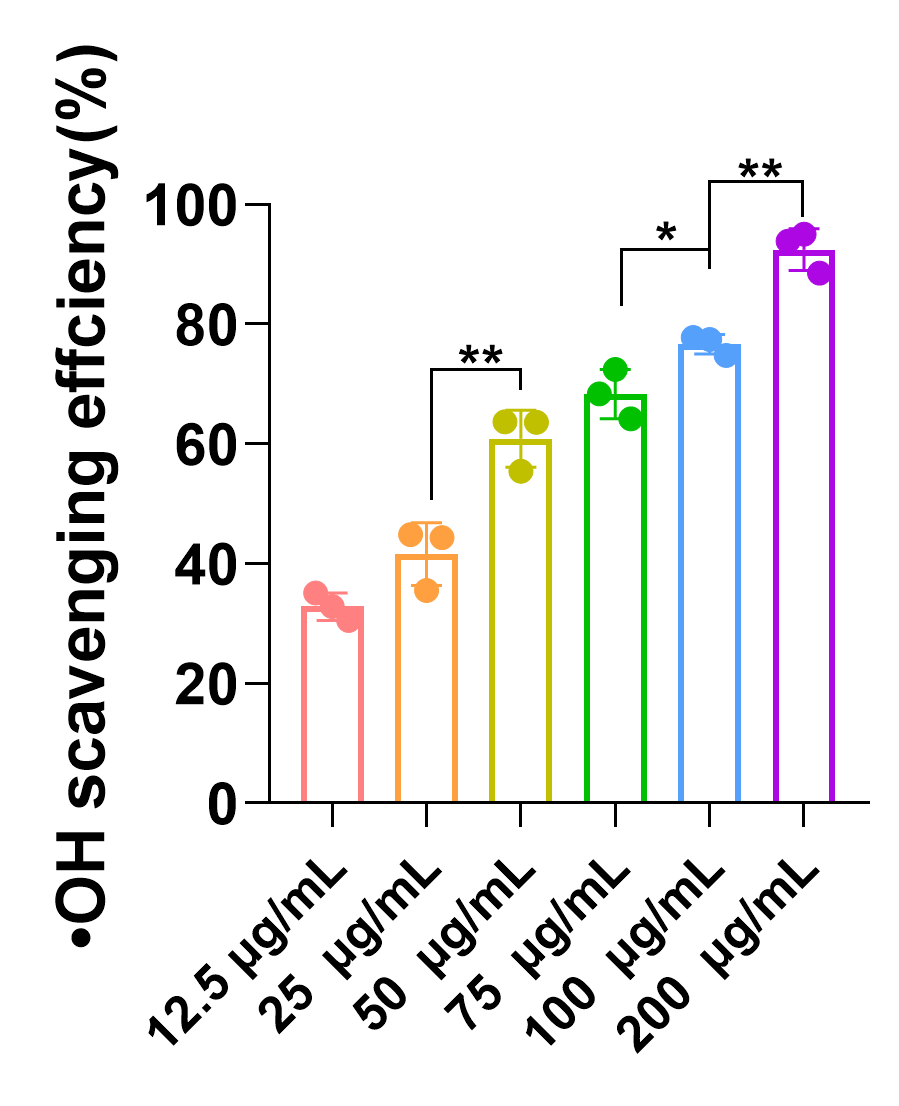
**

***Fig. S6.*** *The calculated scavenging efficiency of* *•OH after incubation with different concentrations of PDA.*

***Fig. S7.*** *UV spectra of H_2_O_2_ in different concentrations and the standard curve of H_2_O_2_ based on the UV values at 240 nm.*

*
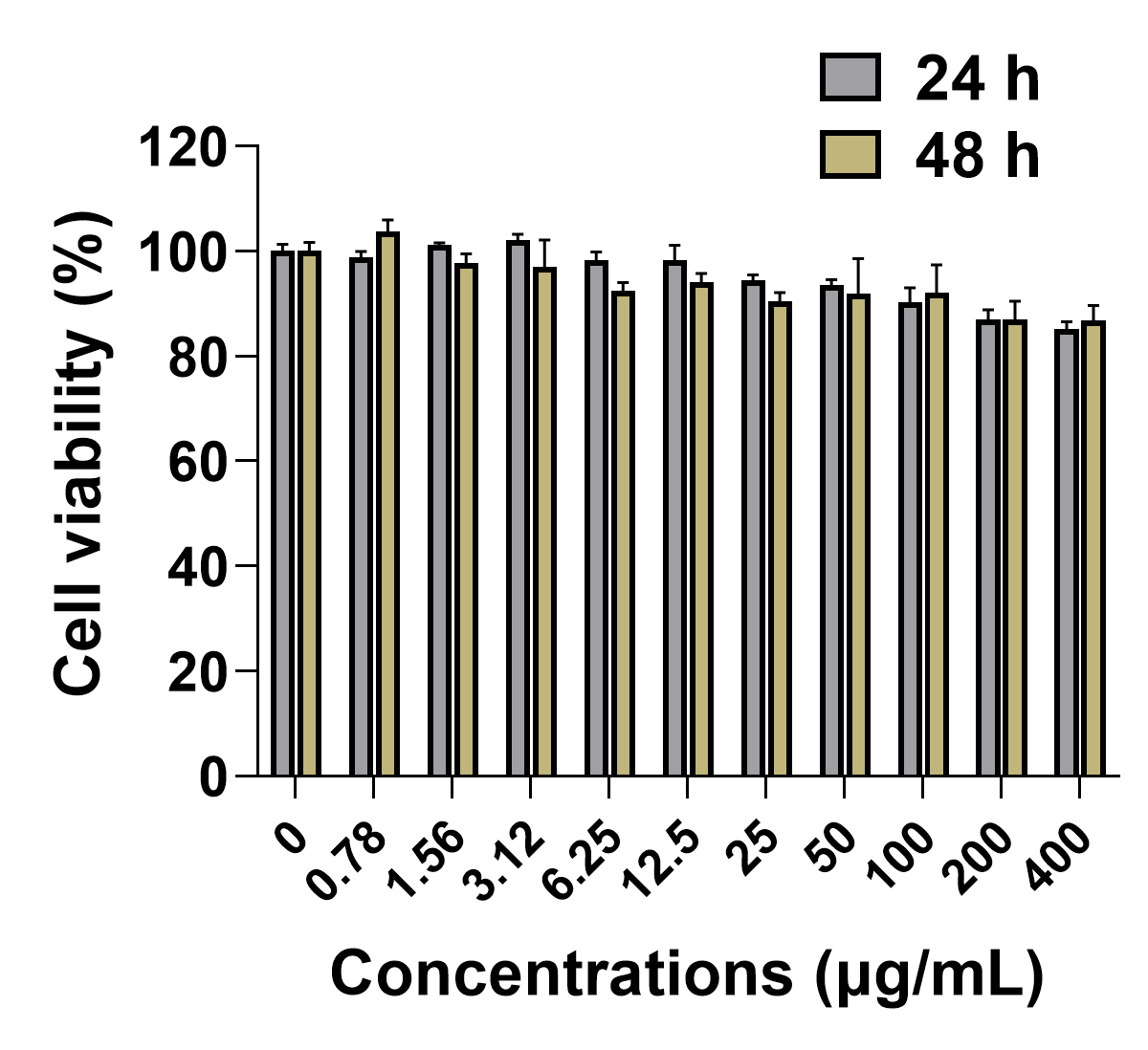
*

***Fig. S8.*** *Cell viability of PC-12 cells after incubation with different concentrations of PDA@K for 24 h and 48 h measured by MTT assay, respectively. Data are presented as mean ± SD, n = 4.*

**
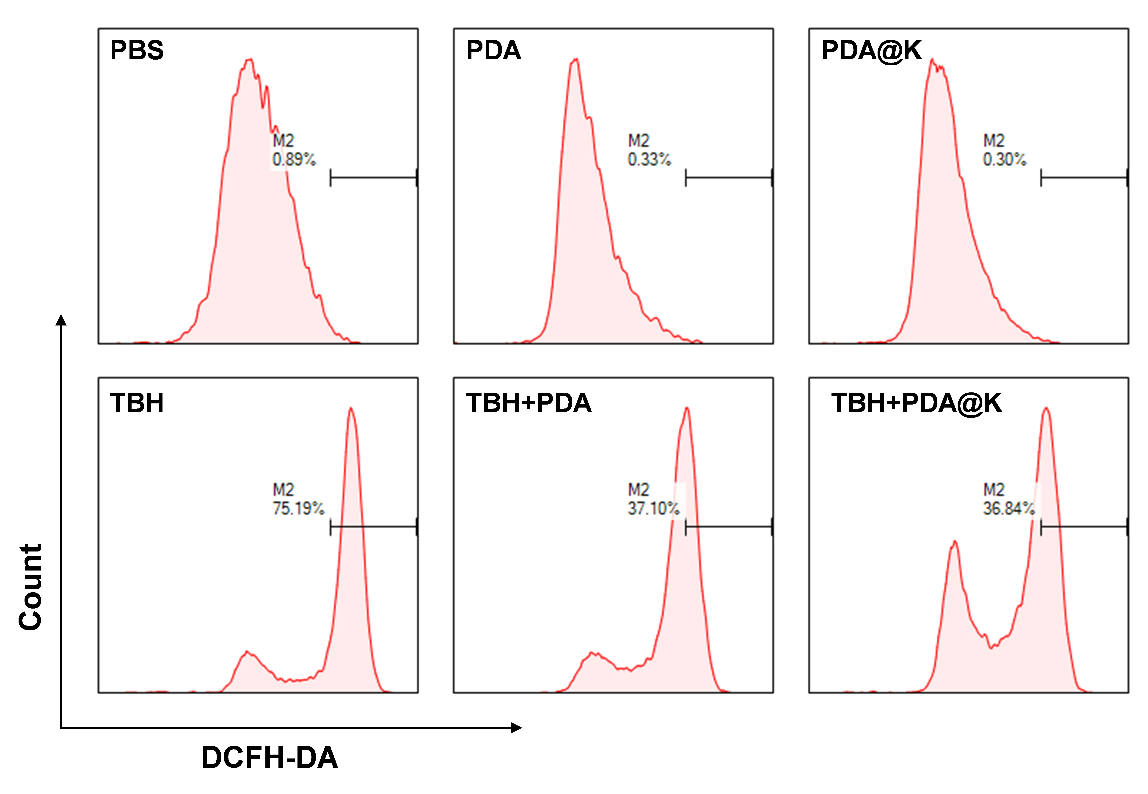
**

***Fig. S9.*** *Representative flow cytometry plots showing fluorescence levels of cells in different experimental conditions.*

**
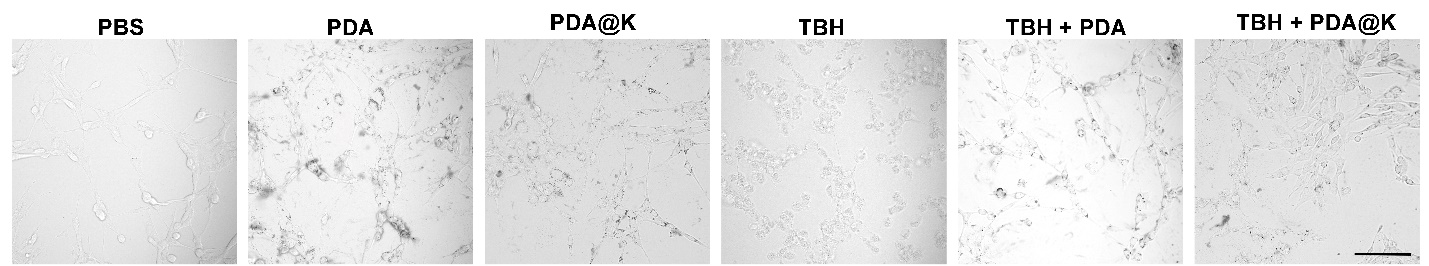
**

***Fig. S10.*** *Changes in cell morphology after 2 h of TBH treatment in the presence and absence of NPs Cells became round and floated up after treatment, while PDA and PDA@K treatment effectively prevented these damages. scale bar: 100 μm.*

***Fig. S11.*** *ThT assay for evaluation of the effect of different concentrations of metal ions on Aβ aggregation. The significant diﬀerence of each group was obtained by comparing with Aβ+PBS group. Data are presented as mean ± SD, n = 3.* **P < 0.05, **P < 0.01, ***P < 0.001.*

***Fig. S12.*** *The effect of serum on inhibitory effect on Aβ aggregation of NPs in the presence of metal ions. NPs were first incubated with serum for 24 h (abbreviated as PDA(s) and PDA@K(s)), followed by incubation with metal ions and Aβ. Data are presented as mean ± SD, n = 3. *P < 0.05, **P < 0.01, ***P < 0.001.*

***Fig. S13.*** *Fluorescence intensity curves of different concentrations of Rhodamine B 5-isothiocyanate (RBITC). The connection efficiency of RBITC is about 10%.*

**
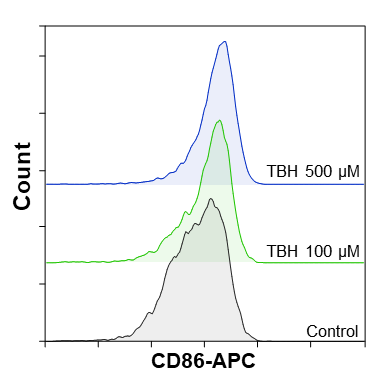
**

***Fig. S14.*** *Validation of microglia polarization toward M1 type under different levels of oxidative stress. Data are presented as mean ± SD, n = 3. *P < 0.05, **P < 0.01, ***P < 0.001.*


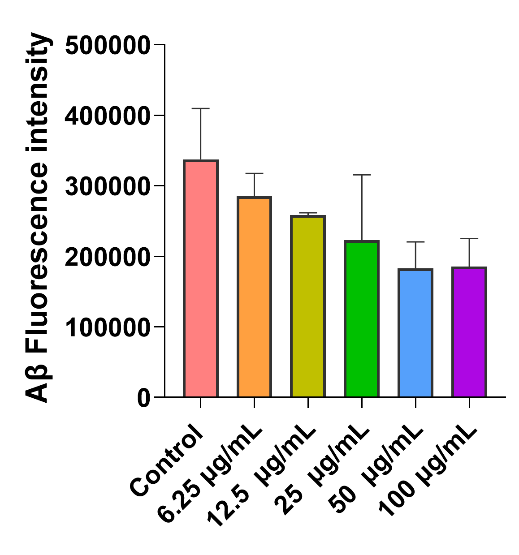


***Fig. S15.*** *Fluorescence intensity of the remaining Aβ in the supernatant after co-incubation of different concentrations of PDA@K with Aβ solutions (4.5 nM). Data are presented as mean ± SD, n = 3.*

**
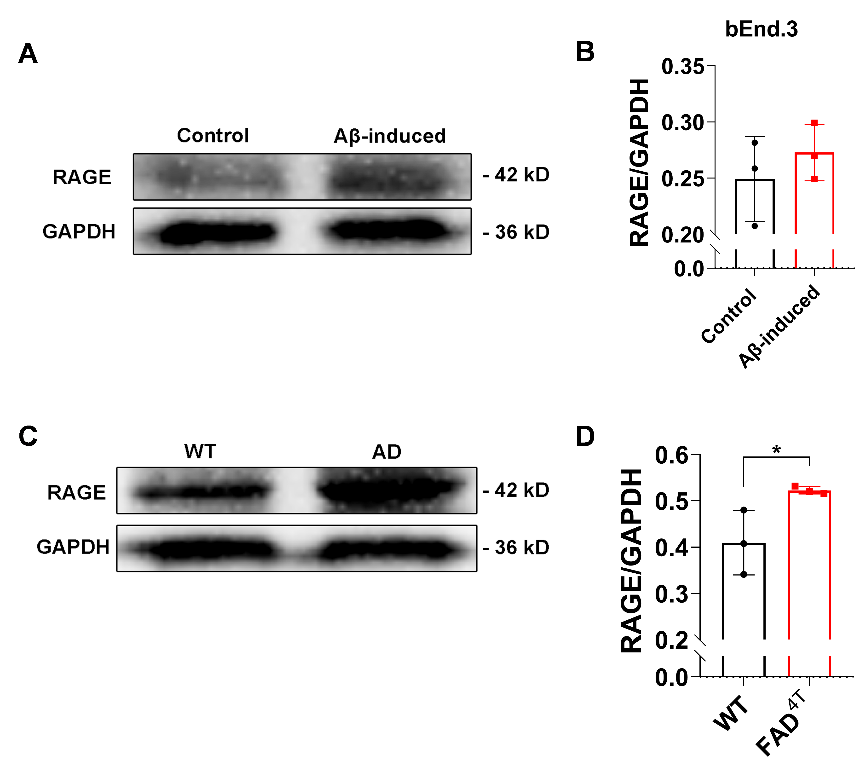
**

***Fig. S16.*** *(A) Western blotting results of RAGE expression in bEnd.3 cells with or without Aβ (10 μM) incubation for 24 h. (B) The corresponding semiquantitative results of A. n= 3 different experiments. (C) Western blotting analysis of RAGE expression in WT and AD mice brains. (D) The corresponding semiquantitative results of C. n= 3 different animals. Data are presented as mean ± SD, *P < 0.05.*


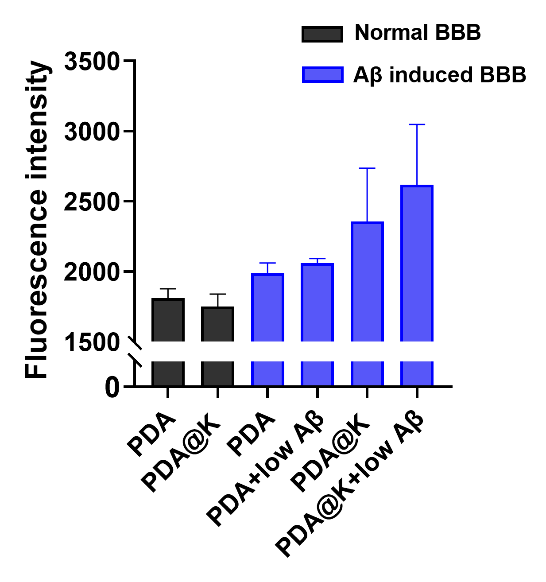


***Fig. S17.*** *Fluorescence intensity of BV2 cells in the lower chamber measured by FCM* *for BBB permeability evaluation. Data are presented as mean ± SD, n = 3.*


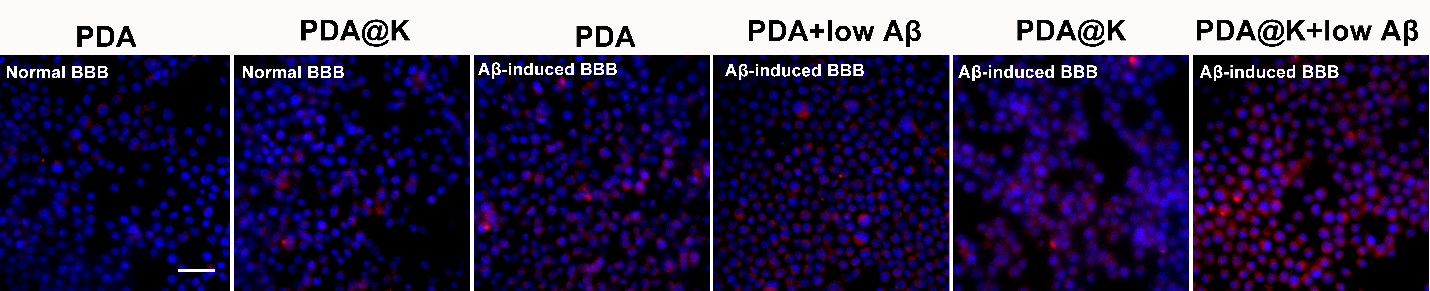


***Fig. S18.*** *CLSM images of BV2 cells in the lower chamber of BBB for BBB permeability evaluation. scale bar, 50* *μm.*

***
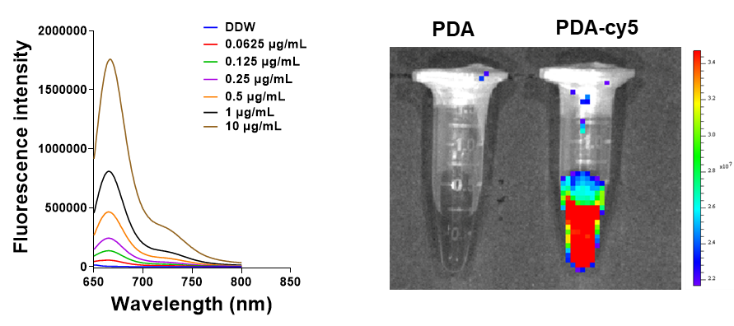
***

***Fig. S19.*** *Fluorescence intensity curves of different concentrations of Cy5. On the right is the fluorescence image of PDA and PDA-cy5 using IVIS Spectrum In Vivo Imaging System.*

**
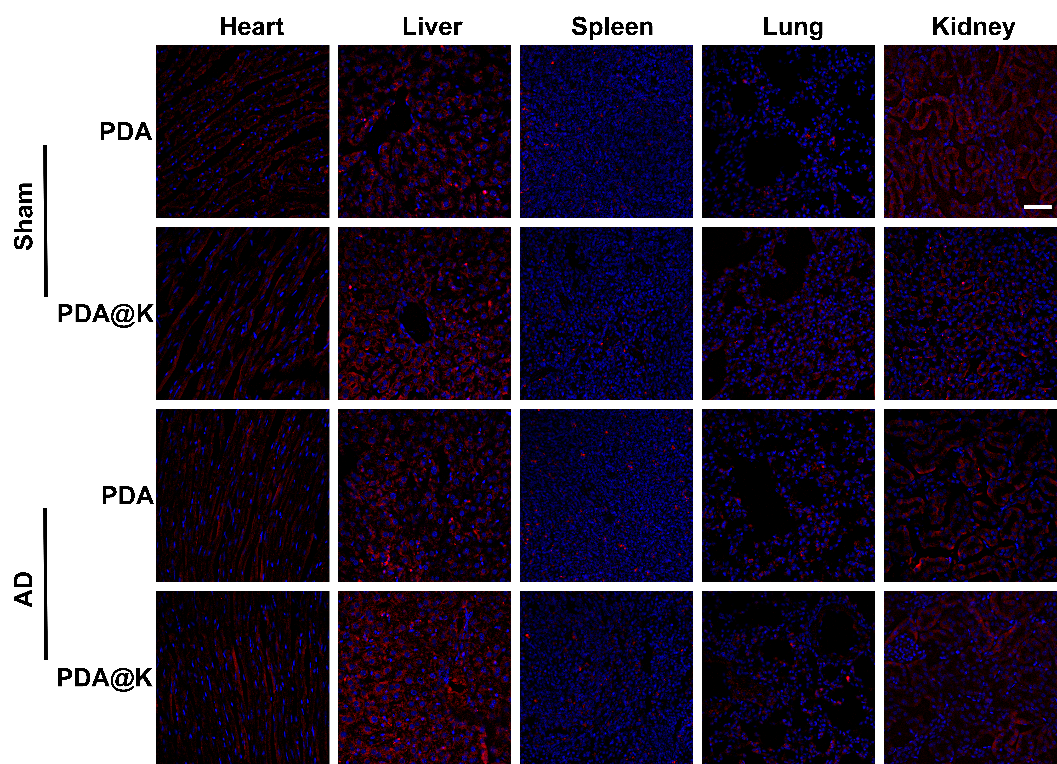
**

***Fig. S20.*** *Fluorescent distribution of PDA and PDA@K in the frozen sections of major organs. Scale bar: 50 μm.*

***Fig. S21.*** *The relative serum concentration of Aβ_42_ of WT, 2 weeks after Aβ-injected model, and FAD^4T^ transgenic mice measured by ELISA.* *Data are presented as mean ± SD, WT: n = 9* *different mice, Aβ-injected mice: n=14 different mice, FAD^4T^ transgenic mice: n=3 different mice.*

**
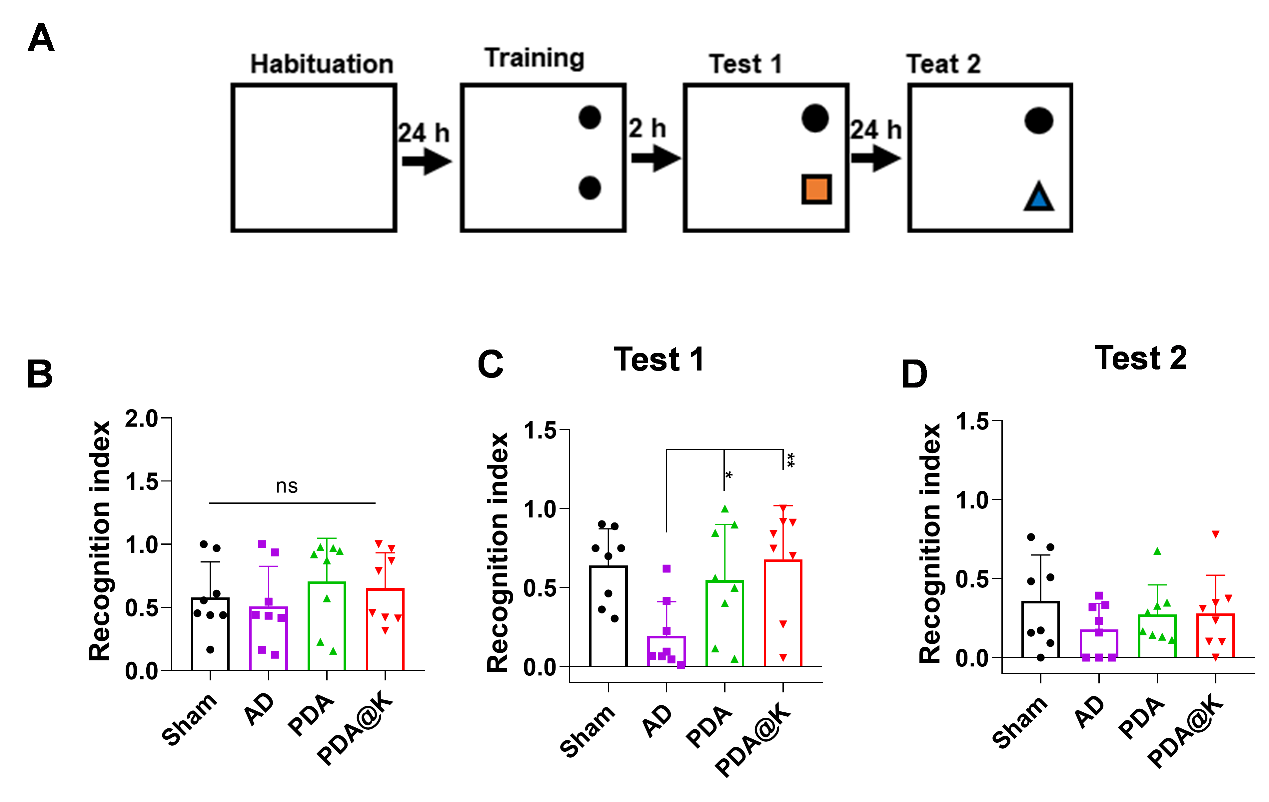
**

***Fig. S22.*** *The novel object recognition (NOR) test of AD mice (Aβ-injected mice) after therapy. (A) The experiment protocol of NOR test. (B-D) The recognition index (RI) calculated in training stage, short-time test (test 1), and long-time test (test 2). Data are presented as mean ± SD, n = 8 mice per group, *P < 0.05, **P < 0.01.*

**
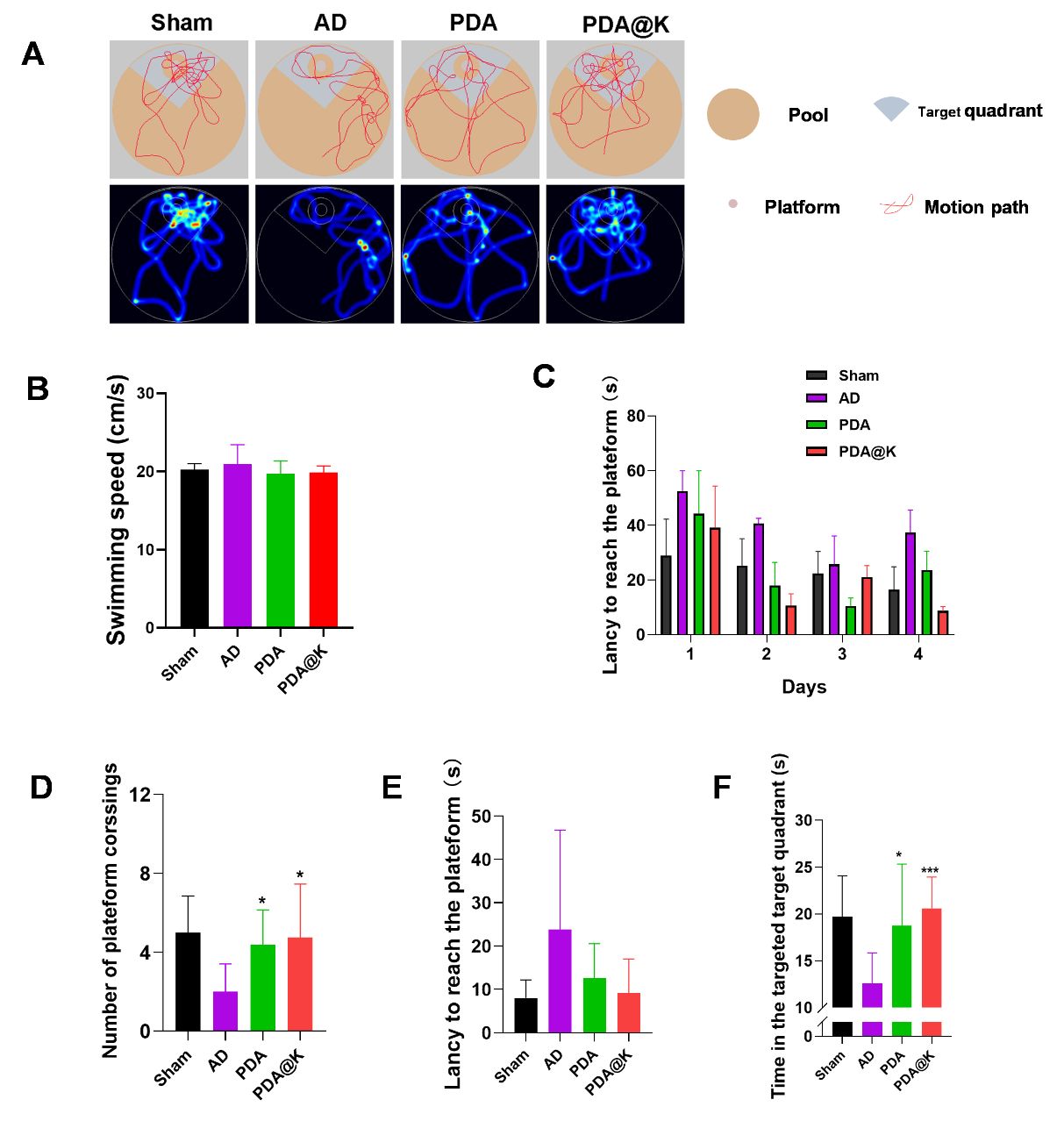
**

***Fig. S23.*** *Morris water maze (MWM) test of AD mice (Aβ-injected mice) after therapy. (A) The searching paths and heat maps of mice in MWM test. (B) Swimming speed and (C) escape latencies during the initial training stage of MWM. Data presented as mean ± SD, n = 8 mice per group. (D) The number of platform crossings, (E) time to reach the target platform, and (F) cumulative time in the quadrant of the platform of mice in diﬀerent groups after treatment. The significant diﬀerence was obtained by comparing each group with sham operation group. Data are presented as mean ± SD, n = 8. *P < 0.05, ***P < 0.001.*


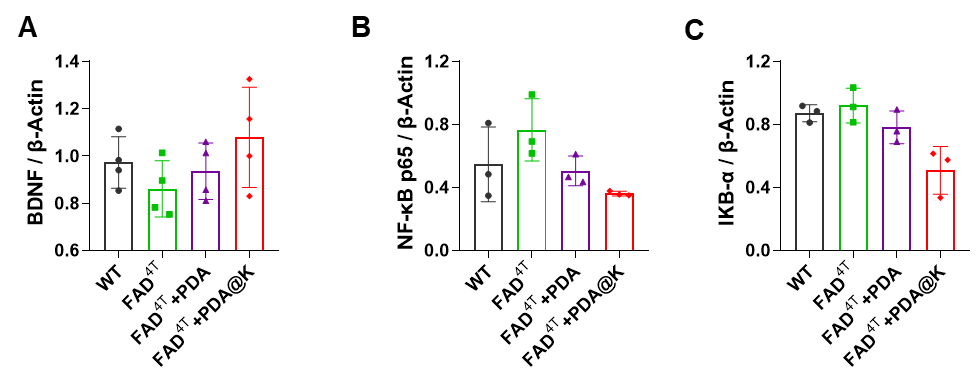


***Fig. S24.****The corresponding semiquantitative results of (A) BDNF (n = 4), (B) NF-κB p65 (n = 3), and (C) IKB-α protein expression (n = 3) in FAD^4T^ mice brain after therapy. Data are presented as mean ± SD.*


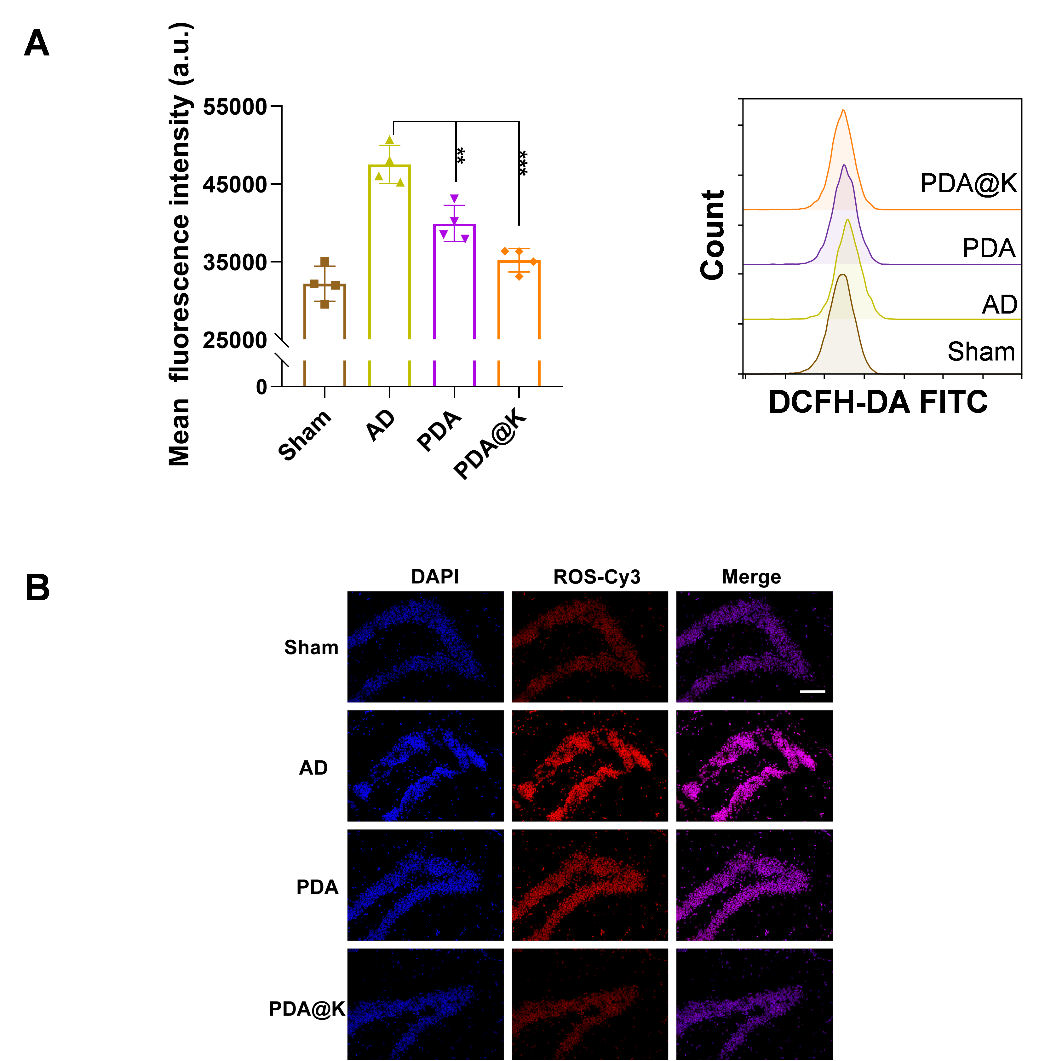


***Fig. S25.*** *(A) Flow cytometry analysis and (B) confocal images for the detection of ROS level in AD mice brain (Aβ-injected mice). Data are presented as mean ± SD, n = 4, **P < 0.01, ***P < 0.001. Scale bar, 100 µm.*


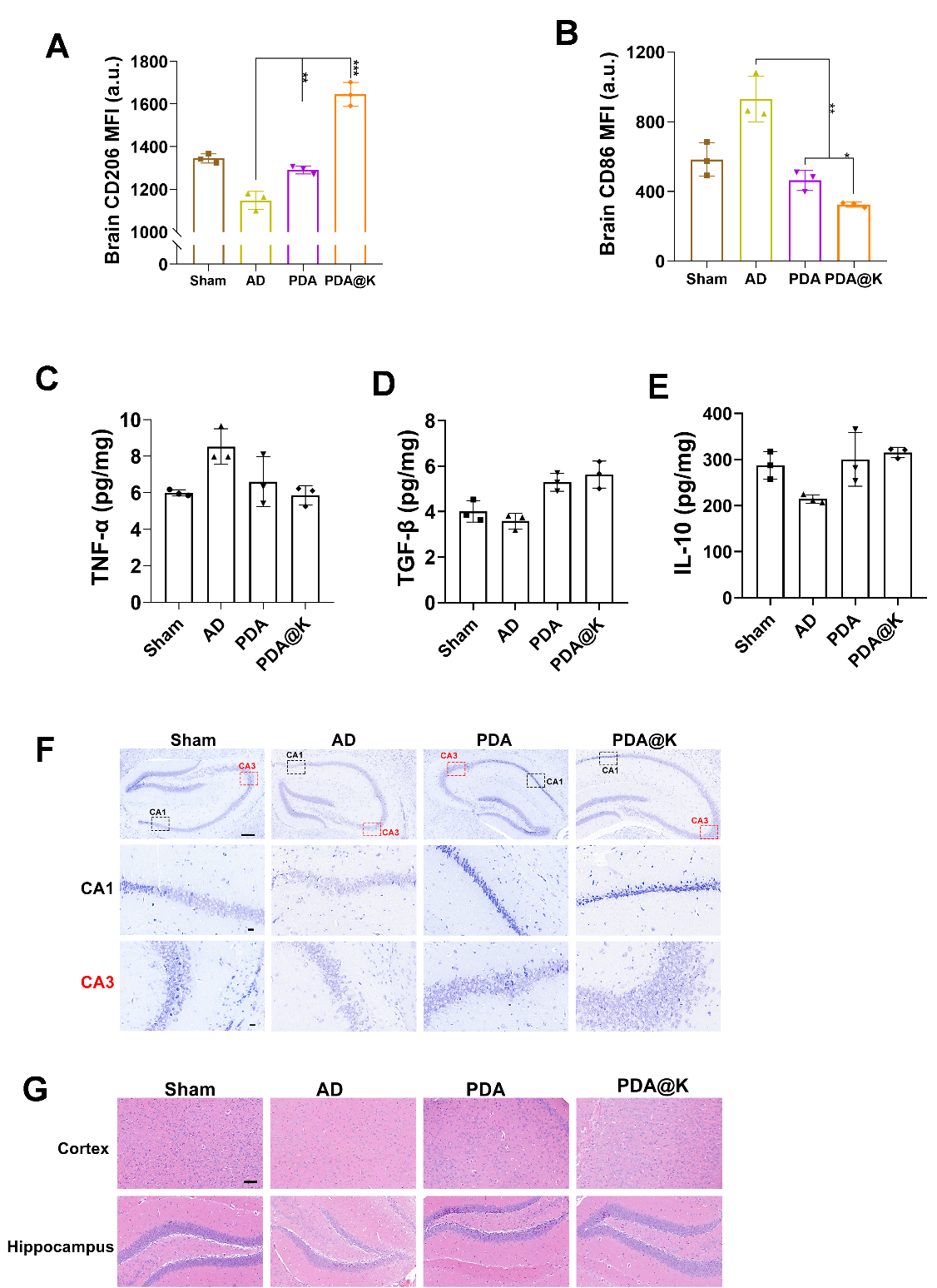


***Fig. S26.*** *(A) CD206 and (B) CD86 expression analysis of AD mice brain (Aβ-injected mice) via flow cytometry after therapy. Data are presented as mean ± SD, n = 3, **P < 0.01, ***P < 0.001. (C-E) ELISA results showing the levels of cytokines of TNF-α, TGF-β, and IL-10 in the brain after treatment. (F) The representative Nissl staining of AD mice brain (Aβ-injected mice) after treatment. The black box circled shows the hippocampal CA1 area, the red shows CA3 area. The first line: scale bar, 200 µm; the last two lines: scale bar, 20 µm. (G) HE staining images of the cortex and hippocampus of Aβ-injected mice brain after treatment. Scale bar, 50 µm.*


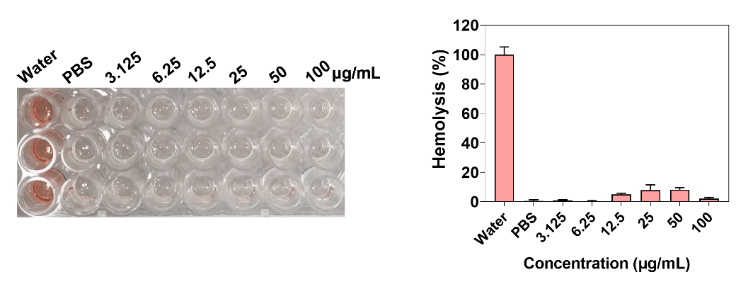


***Fig. S27.*** *Hemolysis test of NPs in different concentrations.*


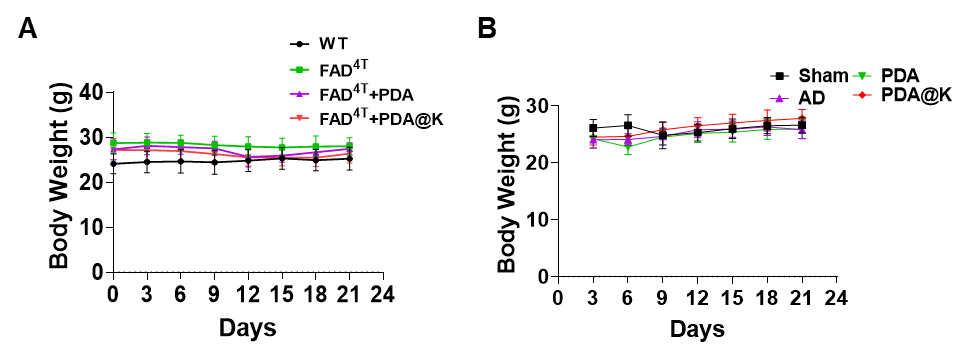


***Fig. S28.*** *Body weight changes of FAD^4T^ transgenic mice and Aβ-injected mice during treatment. (A) Body weight changes of FAD^4T^ transgenic mice during treatment. Data are presented as mean ± SD, n = 10. (B) Body weight changes of Aβ-injected mice during treatment. Data are presented as mean ± SD, n = 8.*


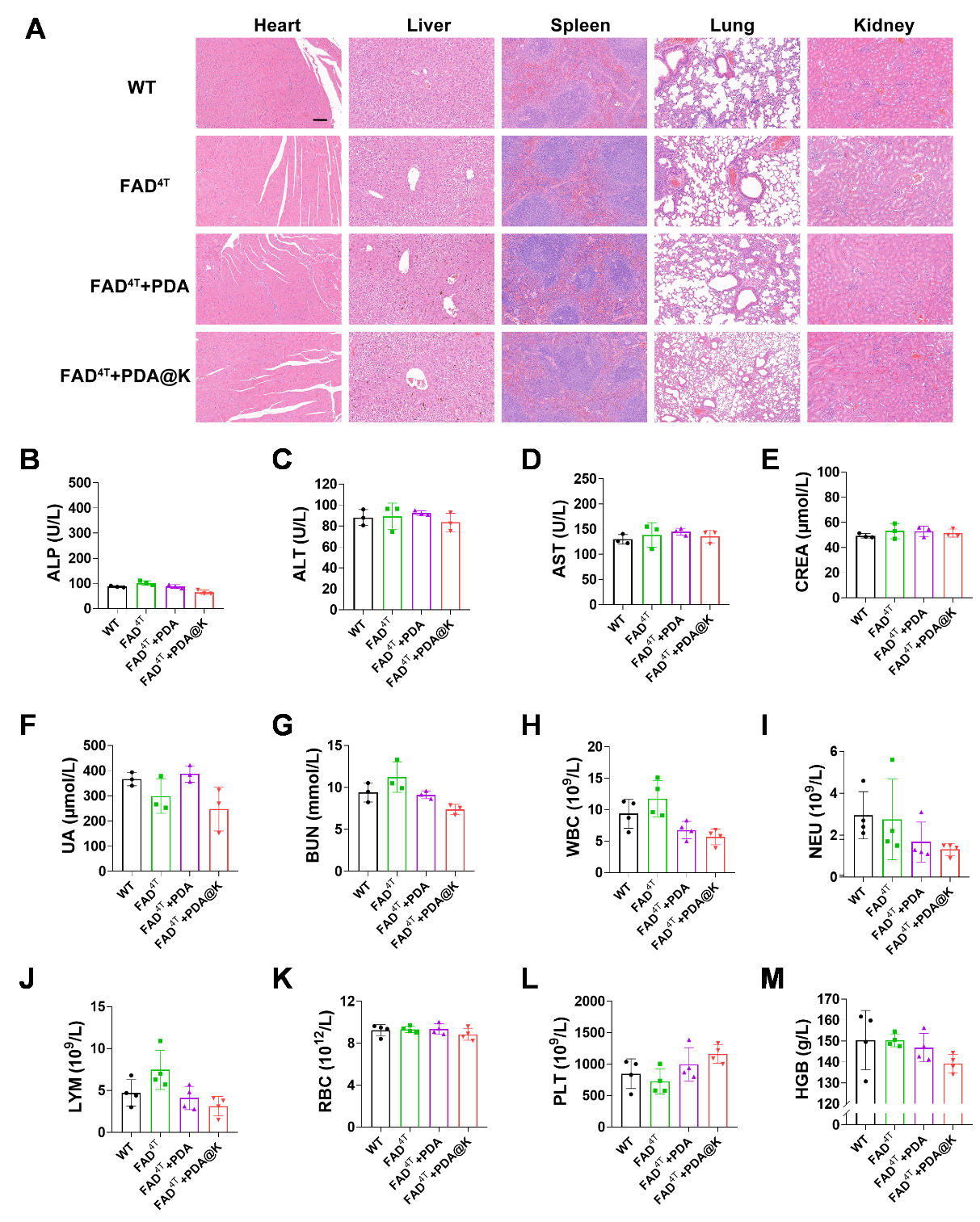


***Fig. S29.*** *Systemic toxicity assessment of NPs in FAD^4T^ transgenic mice after treatment. (A) Representative images for HE staining in major organs from FAD^4T^ transgenic and control WT mice treated with NPs or 5% glucose. Scale bar, 100 µm. (B-G) Blood chemistry examinations after treatment, including alkaline phosphatase (ALP), plasma alanine aminotransferase (ALT), aspartate aminotransferase (AST), creatinine (CREA), uric acid (UA) and plasma urea (BUN). Data are presented as mean ± SD, n = 3. (H-M) Routine blood parameters analysis of leucocytes (WBC), neutrophils (NEU), lymphocytes (LYM), erythrocytes (RBC), platelets (PLT), and hemoglobin (HGB) after treatment. Data are presented as mean ± SD, n = 4.*

**
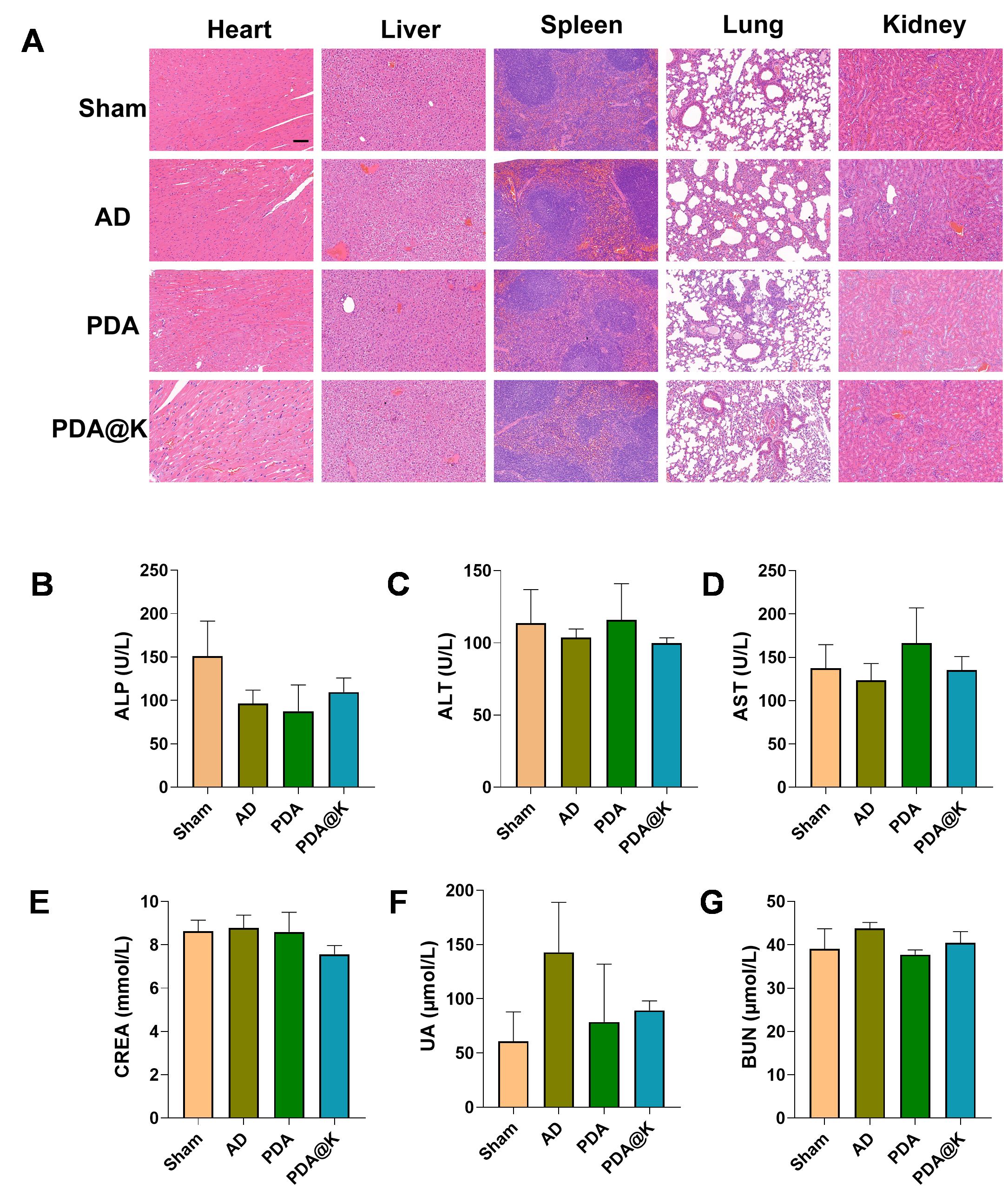
**

***Fig. S30.*** *Systemic toxicity assessment of NPs in Aβ-injected AD mice after treatment. (A) Representative images for HE staining in major organs from* *AD mice (**Aβ-injected mice) and sham operation mice treated with NPs or 5% glucose. Scale bar, 100 µm. (B-G) Blood chemistry examinations of AD mice (Aβ-injected mice) after treatment, including alkaline phosphatase (ALP), plasma alanine aminotransferase (ALT), aspartate aminotransferase (AST), creatinine (CREA), uric acid (UA) and plasma urea (BUN). Data are presented as mean ± SD, n = 3.*
